# DNA fragmentation factor B suppresses interferon to enable cancer persister cell regrowth

**DOI:** 10.1038/s41556-025-01810-x

**Published:** 2025-11-17

**Authors:** August F. Williams, David A. G. Gervasio, Claire E. Turkal, Anna E. Stuhlfire, Michael X. Wang, Brandon E. Mauch, Rhea Plawat, Ariel H. Nguyen, Michelle H. Paw, Mehrshad Hairani, Cooper P. Lathrop, Sophie H. Harris, Jennifer L. Page, Matthew J. Hangauer

**Affiliations:** 1 Department of Dermatology, School of Medicine, University of California San Diego, La Jolla, CA USA; 2 Stem Cell Core, Salk Institute for Biological Studies, La Jolla, CA USA; 3 Moores Cancer Center, University of California San Diego, La Jolla, CA USA; 4 Altman Clinical and Translational Research Institute, University of California San Diego, La Jolla, CA USA

**Keywords:** Apoptosis, Cancer

## Abstract

Oncogene-targeted cancer therapies can provide deep responses but frequently suffer from acquired resistance. Therapeutic approaches to treat tumours that have acquired drug resistance are complicated by continual tumour evolution and multiple co-occurring resistance mechanisms. Rather than treating resistance after it emerges, it may be possible to prevent it by inhibiting the adaptive processes that initiate resistance, but these are poorly understood. Here we report that residual cancer persister cells that survive oncogene-targeted therapy are growth arrested by drug stress-induced intrinsic type I interferon signalling. To escape growth arrest, persister cells leverage apoptotic machinery to transcriptionally suppress interferon-stimulated genes (ISGs). Mechanistically, persister cells sublethally engage apoptotic caspases to activate DNA endonuclease DNA fragmentation factor B (also known as caspase-activated DNase), which induces DNA damage, mutagenesis and stress response factor activating transcription factor 3 (ATF3). ATF3 limits activator protein 1-mediated ISG expression sufficiently to allow persister cell regrowth. Persister cells deficient in DNA fragmentation factor B or ATF3 exhibit high ISG expression and are consequently unable to regrow. Therefore, sublethal apoptotic stress paradoxically promotes the regrowth of residual cancer cells that survive drug treatment.

## Abstract (web summary)

Williams et al. report a growth arrest mechanism in residual cancer persister cells through targeted therapy-induced upregulation of type I interferon signalling, which is negatively regulated by apoptotic DNA endonuclease DFFB to allow tumour relapse.

## Main

**Fig. 1:**
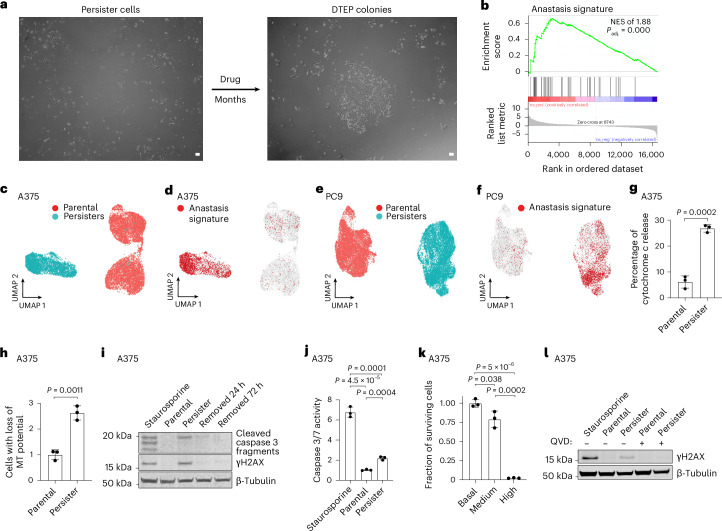
Drug-tolerant persister cells undergo chronic sublethal apoptotic stress-induced DNA damage. **a**, Microscopy of A375 persister and DTEP cells treated with 250 nM dabrafenib and 25 nM trametinib. Scale bars, 100 μm. **b**, Bulk RNA-seq gene set enrichment analysis of the anastasis signature in A375 persister cells. The *P* value was calculated with a permutation test with family-wise error rate correction. **c**–**f**, Single-cell RNA-seq UMAPs of A375 (**c**,**d**) and PC9 (**e**,**f**) parental cells and persister cell populations (**c**,**e**) with enriched anastasis gene set cells highlighted in red (**d**,**f**). PC9 persister cells derived from 2.5 μM erlotinib. **g**, A375 parental and persister cells assessed for mitochondrial release of cytosolic cytochrome *c*. **h**, A375 parental and persister cells assessed for loss of mitochondrial (MT) potential using JC-1 indicator dye by flow cytometry. **i**, A375 persister cells assessed for cleaved caspase 3 and γH2AX either on drug or following 24 or 72 h of drug removal. **j**, Flow cytometry quantification of caspase 3/7 activity reporter geometric means. **k**, A375 persister cells sorted for basal, medium (sublethal) and high (lethal) caspase 3/7 activity, replated in media without drug and assessed for cell viability the following day. **l**, Parental and persister cells assessed for γH2AX with or without cotreatment with 10 μM caspase inhibitor QVD. In **i**, **j** and **l**, parental cells treated for 24 h with 250 nM (**i**,**l**) or for 4 h with 1 μM staurosporine (**j**) were used as a positive control. In **g**, **h**, **j** and **k**, *n* = 3 biological replicates, the mean ± s.d. is shown, and *P* values were calculated with two-tailed Student’s *t*-test. NES, normalized enrichment score. Source data

**Extended Data Fig. 1:**
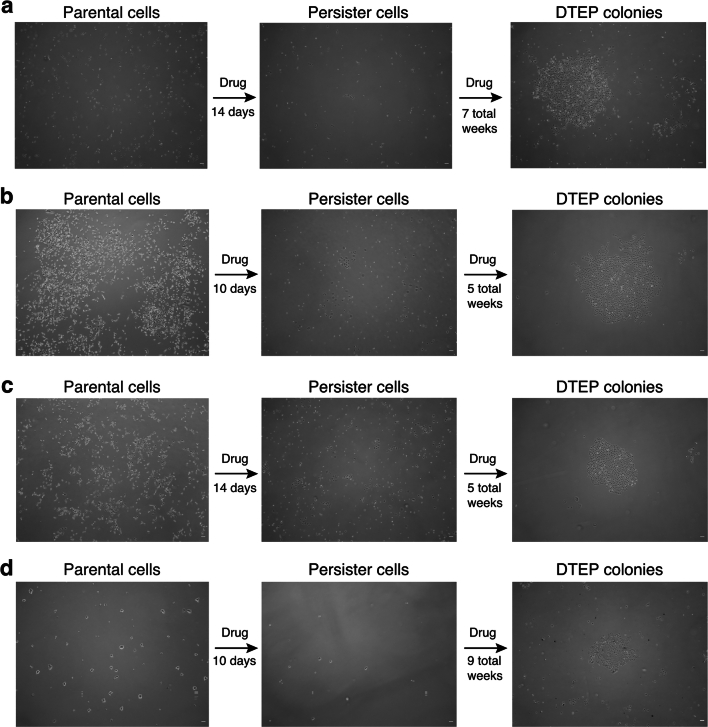
Persister cell and DTEP colony formation models. **a**, A375 BRAF V600E melanoma cells treated with 250 nM dabrafenib and 25 nM trametinib. **b**, PC9 EGFR mutant lung adenocarcinoma cells treated with 2.5 μM erlotinib. **c**, PC9 cells treated with 500 nM osimertinib. **d**, BT474 HER2+ breast cancer cells treated with 2 μM lapatinib. Time shown to DTEP colony formation includes initial persister cell formation treatment period. Scale bar = 100 μm.

To discover adaptive mechanisms that enable cancer cells to acquire drug resistance, we utilized models in which treatment with oncogene-targeted therapies results in death for the majority of cells and effectively purifies a residual population of primarily quiescent cancer persister cells (Extended Data Fig. 1a–d). A minority of persister cells regrow into drug-tolerant expanded persister (DTEP) cell colonies during the first 6 months of treatment, modelling the earliest stage of tumour recurrence (Fig. 1a and Extended Data Fig. 1a–d). Though DTEP colonies have been reported to eventually acquire resistance mutations and become irreversibly drug resistant after 6 months or more of treatment^1,2^, it is not known whether mutations contribute to initial DTEP colony formation.

To gain insight into the transition from persister to DTEP cells (Fig. 1a), we performed RNA sequencing (RNA-seq) on targeted therapy-treated persister cells. Beyond previously described persister cell signatures^1,3–7^, we observed an enrichment in an ‘anastasis’ signature (Fig. 1b–f and Supplementary Tables 1–3). Anastasis is the phenomenon wherein cells recover from brief periods of apoptotic stress, and this can result in DNA damage, mutagenesis and transformation^8^. Similarly, ‘flatliner’ cells which survive transient mitochondrial outer membrane permeabilization (MOMP), a key initiating step toward apoptosis, were recently shown to have increased potential to form persister cells and also to metastasize^9^. The melanoma cells which undergo ‘failed apoptosis’ also gain migration and invasion properties^10^. Therefore, brief encounters with apoptotic stress can have protumour effects. Whether cancer cells can also tolerate weeks or months of continuous apoptotic stress such as in the context of acquired drug resistance is unknown.

## Results

### Persister cells undergo chronic sublethal apoptotic signalling

**Extended Data Fig. 2:**
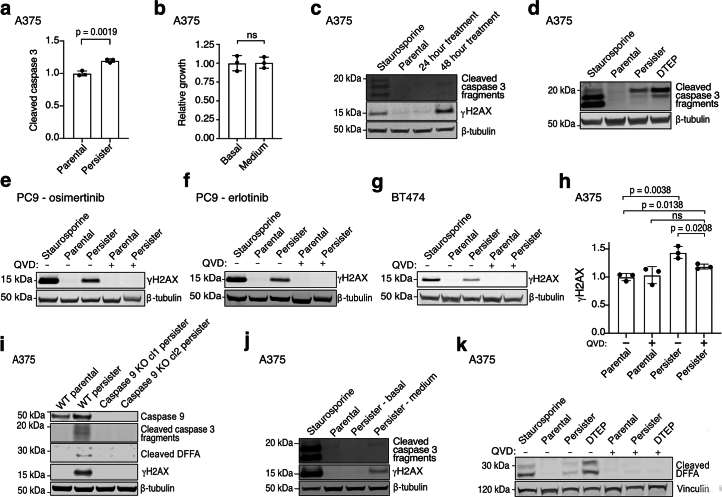
Persister cells undergo chronic drug-induced sublethal apoptotic stress that causes DNA damage. **a**, Quantification of cleaved caspase 3 from flow cytometry of live-gated cells. **b**, Relative growth in drug-free media for 6 days of A375 persister cells after sorting for basal or medium (sublethal elevated) caspase 3/7 activity. See Supplementary Fig. 5 for sorting strategy. **c**, A375 cells treated with 250 nM dabrafenib and 25 nM trametinib for 24 or 48 h and assessed for cleaved caspase 3 or DNA damage marker γH2AX. **d**, A375 parental, persister, and DTEP cells derived from 7 weeks of treatment with 250 nM dabrafenib and 25 nM trametinib analyzed for cleaved caspase 3. **e-h**, Parental and persister cells assessed for γH2AX with and without treatment with 10 μM caspase inhibitor QVD. **e**, PC9 cells treated with 500 nM osimertinib. **f**, PC9 cells treated with 2.5 μM erlotinib. **g**, BT474 cells treated with 500 nM lapatinib. **h**, A375 parental and persister cells assessed by flow cytometry for γH2AX. **i**, A375 WT and caspase 9 KO clones analyzed for caspase 9, cleaved caspase 3, cleaved DFFA, and γH2AX. **j**, A375 persister cells sorted by caspase 3/7 activity levels and assessed for cleaved caspase 3 and γH2AX. **k**, A375 persister and DTEP cell caspase-dependent cleaved DFFA. 250 nM (**c**,**d**,**j**,**k**), 500 nM (**e**,**f**), or 750 nM (**g**) staurosporine treatment for 24 h was used as a positive control. For **a**,**b**,**h**, *n* = 3 biological replicates, mean $\pm$ s.d.; two-tailed Student’s t-test; ns, not significant. Source data

We found that persister cells exhibit partial mitochondrial cytochrome *c* release (Fig. 1g and Supplementary Fig. 1) and loss of mitochondrial potential (Fig. 1h and Supplementary Fig. 2) indicative of sublethal MOMP. Persister cells also have partially cleaved apoptotic executioner caspase 3 which is at a level below cells treated with a lethal exposure of apoptosis-inducer staurosporine, consistent with sublethal caspase activation (Fig. 1i, Extended Data Fig. 2a and Supplementary Fig. 3). Furthermore, using a fluorescent caspase 3/7 activity sensor, we observed an increase in caspase activity in live persister cells (Fig. 1j and Supplementary Fig. 4), and sorted persister cells with increased levels of caspase 3/7 activity were capable of regrowth upon drug removal, whereas cells with very high caspase activity were not, demonstrating that persister cells remain viable with apoptotic caspase activity (Fig. 1k and Extended Data Fig. 2b). This is consistent with a recent study showing that cells can recover from intermediate but not high apoptotic caspase 3 activity^11^. In addition, though cancer cell lines can have spontaneous low level apoptotic signalling^12^, we found that persister cell apoptotic signalling is drug-induced because apoptotic signalling markers are absent before treatment, induced early during drug exposure, maintained throughout months of treatment and dissipated within 24 h of drug removal (Fig. 1i and Extended Data Fig. 2c,d). Therefore, persister cells experience drug stress-induced apoptotic signalling throughout drug exposure yet remain alive.

### DFFB induces DNA damage in persister cells

In response to a variety of acute stresses, apoptotic caspases can promote DNA damage by activating apoptotic DNA endonucleases^8,13–15^. We tested whether apoptotic caspases also induce DNA damage in persister cells and found that the pan-caspase inhibitor quinoline-Val-Asp-difluorophenoxymethylketone (QVD) blocked persister cell DNA damage across multiple tumour types treated with targeted therapies (Fig. 1l, Extended Data Fig. 2e–h and Supplementary Fig. 5). Similarly, caspase 9-knockout (KO) persister cells, which do not undergo drug-induced caspase 3 cleavage, fail to acquire DNA damage (Extended Data Fig. 2i). We also found that persister cells sorted for increased caspase 3/7 activity have increased DNA damage compared with persister cells without increased caspase activity (Extended Data Fig. 2j). Therefore, targeted therapy drugs induce apoptotic caspase-dependent DNA damage in persister cells.

**Fig. 2:**
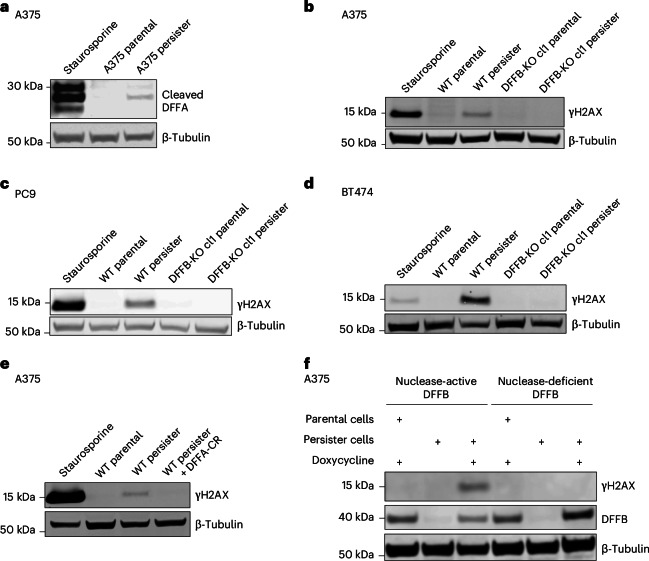
Apoptotic DNase DFFB is the primary DNA damage source in cancer persister cells. **a**, A375 persister cells exhibit cleaved DFFA, indicating DFFB activation. **b**–**d**, Persister cell DNA damage is absent in A375 (**b**), PC9 (500 nM osimertinib) (**c**) and BT474 (500 nM lapatinib) (**d**) DFFB LOF persister cells. In **a**–**e**, 250 nM (**a**,**b**,**e**), 500 nM (**c**), or 750 nM (**d**) staurosporine treatment for 24 h was used as a positive control. **e**, Persister cell DNA damage is absent in A375 cells with the ectopically expressed cleavage-resistant DFFA variant (DFFA-CR). **f**, Doxycycline-inducible re-expression of nuclease-active DFFB, but not nuclease-dead DFFB, in DFFB-KO A375 persister cells restores drug-induced DNA damage. cl, clone; WT, wild-type.

**Extended Data Fig. 3:**
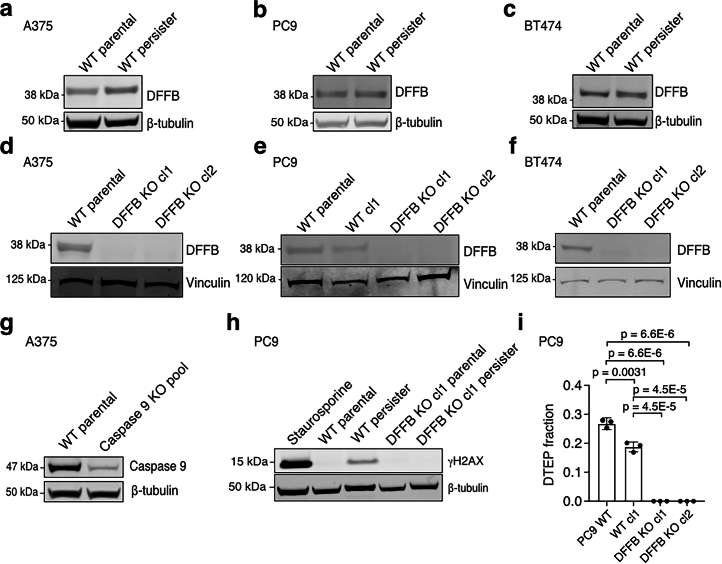
CRISPR knockout, DNA damage and DTEP formation data. **a-c**, DFFB expression in parental and persister cells. **a**, A375 cells treated with 250 nM dabrafenib and 25 nM trametinib. **b**, PC9 cells treated with 2.5 μM erlotinib. **c**, BT474 cells treated with 500 nM lapatinib. **d-f**, DFFB loss of expression in CRISPR KO clones. cl, clone. **d**, A375 DFFB KO clones. **e**, PC9 DFFB KO clones. WT cl1 is a control WT clone which failed to lose DFFB expression during CRISPR editing. **f**, BT474 DFFB KO clones. **g**, Caspase 9 expression in CRISPR-mediated caspase 9-depleted (pooled KO) A375 cells used in Fig. 4g. **h**, PC9 cells treated with 2.5 μM erlotinib were assessed for DNA damage marker γH2AX. Parental cells were treated for 24 h with 500 nM staurosporine as a positive control. **i**, PC9 cells treated with 2.5 μM erlotinib for 5 weeks were analyzed for DTEP formation. *n* = 3 biological replicates, mean $\pm$ s.d.; two-tailed Student’s t-test. Source data

During apoptosis, DNA endonuclease DNA fragmentation factor B (DFFB) is activated by caspase 3-mediated proteolytic cleavage of DFFB chaperone and inhibitor protein DNA fragmentation factor A (DFFA, also known as ICAD or DFF45), enabling DFFB to form a nuclease active dimer^16^. Activated DFFB induces DNA breaks, promoting the extensive fragmentation of chromosomal DNA during apoptotic cell death^17–19^, but in response to transient sublethal apoptotic stress, DFFB can also induce acute DNA damage^13–15,20^. We explored whether the chronic apoptotic stress we observed in persister cells results in DFFB-mediated persistent DNA damage. Consistent with the activation of DFFB, we observed the cleavage of DFFA in persister cells (Fig. 2a) which is dependent on caspases (Extended Data Fig. 2i,k). DFFA cleavage was also observed in DTEP cells, indicating continuous DFFB activation throughout DTEP colony formation (Extended Data Fig. 2k). To test whether DFFB functionally contributes to DNA damage, we generated DFFB loss-of-function (LOF) models including CRISPR-mediated DFFB-KO cells (Extended Data Fig. 3a–g) and cells with ectopically expressed non-cleavable mutant DFFA, which is resistant to caspase-mediated cleavage and blocks endogenous DFFB activity^21^. We found that drug-induced DNA damage was absent in all DFFB LOF persister cell models (Fig. 2b–e and Extended Data Fig. 3h), and the re-expression of wild-type DFFB, but not nuclease activity-deficient mutant DFFB^22^, restored drug-induced DNA damage in DFFB-KO persister cells (Fig. 2f). Therefore, DFFB endonuclease activity is the primary source of drug stress-induced DNA damage in targeted therapy-treated persister cells.

### DFFB is required for persister cell regrowth and tumour relapse

**Fig. 3:**
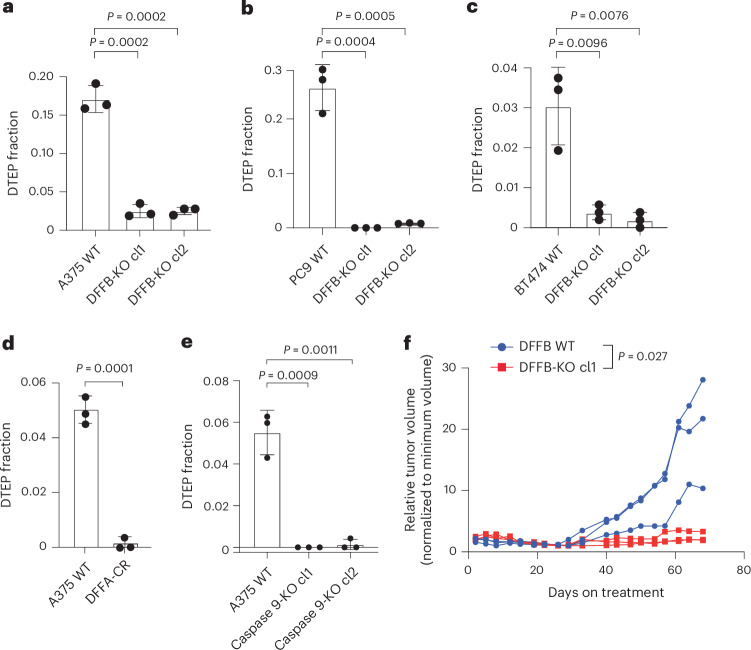
DFFB mediates cancer persister cell regrowth. **a**–**e**, Measurement of the fraction of cells which regrow into DTEP colonies during treatment; A375 melanoma cells treated with 250 nM dabrafenib and 25 nM trametinib comparing wild-type to DFFB-KO (**a**), DFFA-CR-expressing (**d**), or caspase 9-KO (**e**) cells; PC9 lung cancer cells treated with 300 nM osimertinib comparing wild-type to DFFB-KO cells (**b;**) and BT474 breast cancer cells treated with 2 μM lapatinib comparing wild-type to DFFB-KO cells (**c**). *n* = 3 biological replicates; mean ± s.d.; two-tailed Student’s *t*-test. **f**, Tumour volume of A375 xenograft tumours in mice treated with dabrafenib and trametinib. Data are normalized to the minimum volume of each tumour during treatment; *n* = 3 biological replicates; the individual measurements are displayed. The two-tailed Student’s *t*-test was calculated on the mean of wild-type versus the mean of DFFB-KO. Source data

**Extended Data Fig. 4:**
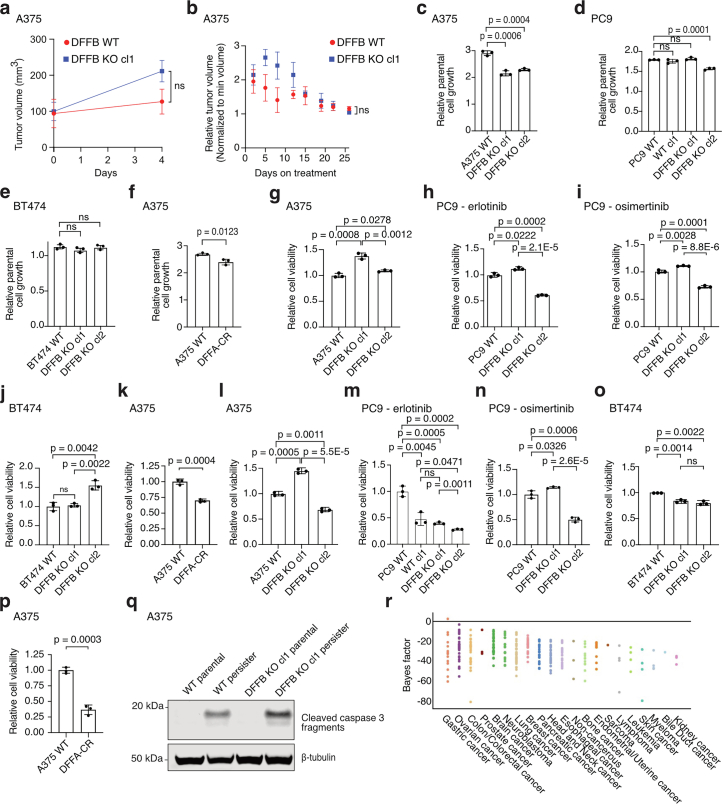
DFFB deficiency does not affect parental tumour cell proliferation or initial drug response. **a-b**, A375 DFFB KO cells form tumours and respond to drug in vivo similarly to WT cells. cl, clone. **a**, A375 xenograft tumour formation and **b**, initial drug response to dabrafenib and trametinib in mice. n = 6 biological replicates; mean ± s.d. is shown; P values calculated with two-tailed Student’s t-test; ns, not significant. **c-f**, Clonal variability in parental cell growth rates in cell culture is independent of DFFB WT or loss of function (LOF) status. DFFA-CR, cleavage resistant DFFA. **g-k**, WT and DFFB LOF cell initial 3 day drug treatment response. **g**,**k**, A375 cells treated with 250 nM dabrafenib and 25 nM trametinib. **h**, PC9 cells treated with 2.5 μM erlotinib. **i**, PC9 cells treated with 500 nM osimertinib. **j**, BT474 cells treated with 2 μM lapatinib. **l-p**, WT and DFFB LOF persister cell formation. **l**,**p**, A375 cells treated with dabrafenib and trametinib. **m**, PC9 cells treated with erlotinib. **n**, PC9 cells treated with 500 nM osimertinib. **o**, BT474 cells treated with 2 μM lapatinib. For **c-p**, *n* = 3 biological replicates; mean $\pm$ s.d. is shown; P values calculated with two-tailed Student’s t-test. ns, not significant. **q**, Cleaved caspase 3 analyzed in A375 WT and DFFB KO parental and persister cells. **r**, DFFB essentiality in CRISPR screen data from the PICKLES database^71^. Negative scores reflect non-essentiality. Source data

Given prior observations that DFFB activation can promote transformation^14,23^, we explored whether chronic DFFB-induced DNA damage may promote persister cell escape from growth arrest to enable regrowth into DTEP colonies. Indeed, we found that DFFB LOF persister cells were severly hindered in their ability to form DTEP colonies across all tested tumour types and treatments (Fig. 3a–e and Extended Data Fig. 3i). We then tested whether DFFB is required for acquired resistance in vivo. Mice bearing A375 wild-type DFFB or DFFB-KO melanoma tumours, which formed similarly (Extended Data Fig. 4a), were treated with dabrafenib and trametinib. Both wild-type and DFFB-KO tumours initially responded to therapy and shrunk to a similar minimal residual volume (Extended Data Fig. 4b). However, whereas wild-type DFFB tumours regrew during further treatment, DFFB-KO tumours remained in a regressed state and failed to regrow (Fig. 3f). These observations are independent of tumour cell viability, initial drug response or persister cell formation, which were each found to be unaffected by DFFB deficiency (Extended Data Fig. 4). Therefore, DFFB is specifically required for persister cells and residual tumours to regrow during drug treatment.

### DFFB induces mutagenesis in persister cells

**Extended Data Fig. 5:**
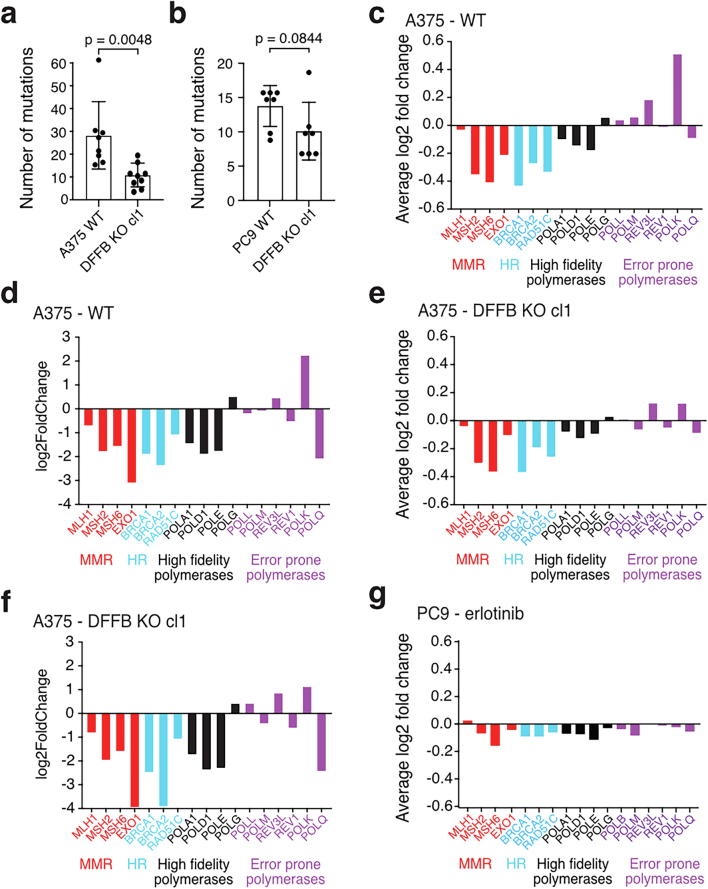
DFFB contributes to persister cell mutagenesis. **a**,**b**, Whole exome sequencing analysis of mutations acquired by A375 cells during 7 weeks of dabrafenib and trametinib treatment (**a**) and PC9 cells during 10 weeks of 2.5 μM erlotinib treatment (**b**). **a**, *n* = 8 WT biological replicates and 9 DFFB KO biological replicates. **b**, *n* = 7 WT and 7 DFFB KO biological replicates. **a**,**b**, Mean $\pm$ s.d.; two-tailed Student’s t-test. **c-g**, Comparison of gene expression changes associated with stress-induced mutagenesis including downregulated repair genes, downregulated high fidelity polymerases, and upregulated error prone polymerases^3^, in persister versus parental cells. **c-f**, A375 persister cells derived from 250 nM dabrafenib and 25 nM trametinib. **c,d**, A375 WT cells analyzed with (**c**) scRNAseq and (**d**) bulk RNAseq. **e,f**, A375 DFFB KO cells analyzed with (**e**) scRNAseq and (**f**) bulk RNAseq. cl, clone. **g**, PC9 persister cells treated with 2.5 μM erlotinib and analyzed with scRNAseq. Mismatch repair (MMR); homology-directed repair (HR). Source data

We next explored the mechanism by which DFFB enables persister cell regrowth. One possible mechanism is by inducing mutagenesis and the acquisition of resistance mutations because DFFB has been reported to promote mutagenesis during acute apoptotic stress^13,23,24^. To test this possibility, we performed whole-exome sequencing on the wild-type DFFB and DFFB-KO A375 cells subjected to 7 weeks of dabrafenib and trametinib treatment, which revealed a variety of acquired mutations, supporting recent reports of persister cell stress-induced mutagenesis^3,25^ (Supplementary Table 4). Consistent with DFFB-mediated mutagenesis, we found that A375 DFFB-KO cells had fewer than half as many acquired mutations as wild-type cells (Extended Data Fig. 5a). An analysis of PC9 cells subjected to 10 weeks of erlotinib treatment similarly revealed that PC9 DFFB-KO cells acquired fewer mutations than wild-type cells (Extended Data Fig. 5b and Supplementary Table 5). However, the magnitude of the contribution of DFFB to acquired mutations was less in PC9, potentially because PC9 persister cells also undergo substantial APOBEC3-mediated mutagenesis^25^ and lack the downregulation of DNA repair genes or upregulation of error prone DNA polymerases, a previously reported feature of stress-induced mutagenesis^7^ (Extended Data Fig. 5c–g). These data suggest DFFB is one of multiple sources of mutagenesis in persister cells. However, there were no known resistance conferring mutations found among the acquired mutations (Supplementary Tables 4 and 5). We therefore searched for an alternative non-mutational mechanism by which DFFB promotes persister cell regrowth.

### DFFB suppresses IFN signalling to enable persister cell regrowth

**Fig. 4:**
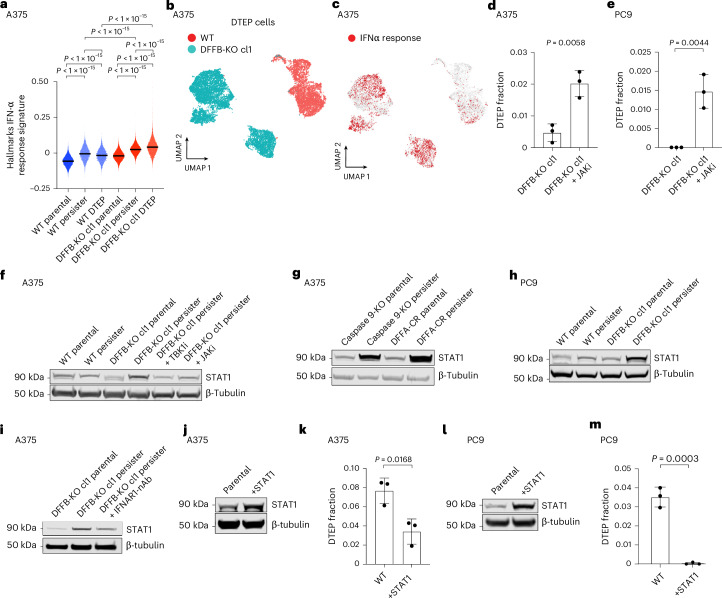
DFFB suppresses IFN signalling to allow persister cell regrowth. **a**, Single-cell RNA-seq Hallmarks IFNα response gene set signature scores in A375 wild-type DFFB and DFFB-KO parental cells and drug-treated cells at the persister and DTEP timepoints. *P* values were calculated with the two-sided Mann–Whitney test. **b**,**c**, Single-cell RNA-seq UMAP of similar numbers of A375 wild-type and DFFB-KO cells at 9 weeks of drug treatment (**b**) with the enriched Hallmarks IFNα response gene set highlighted in red (**c**). **d**,**e**, Measurement of the fraction of cells which regrew into DTEP colonies during treatment: A375 DFFB-KO cells treated with 250 nM dabrafenib and 25 nM trametinib for 2 weeks to derive persister cells, then 1 μM JAK inhibitor (JAKi) ruxolitinib was added and all three drugs were maintained for five more weeks (**d**), and PC9 DFFB-KO cells were treated with 300 nM osimertinib and 5 μM ruxolitinib for 5 weeks (**e**). **f**, Total STAT1 levels in A375 persister cells cotreated for 14 days with 1 μM TBK1 inhibitor MRT67307 or 1 μM JAKi. **g**, A375 caspase 9-depleted (pooled KO) and DFFA-CR-expressing parental and persister cells analysed for STAT1. **h**, PC9 wild-type and DFFB-KO persister cells treated with 500 nM osimertinib analysed for STAT1. **i**, The A375 DFFB-KO cells treated for 7 days with 250 nM dabrafenib and 25 nM trametinib with and without 1.5 μM of IFNα receptor 1 neutralizing antibody (IFNAR1-nAb) and analysed for total STAT1 expression. **j**,**k**, A375 wild-type and STAT1-overexpressing cells (**j**) treated with 250 nM dabrafenib and 25 nM trametinib and analysed for DTEP colony formation (**k**). **l**,**m**, PC9 wild-type and STAT1-overexpressing cells (**l**) treated with 300 nM osimertinib and analysed for DTEP colony formation (**m**). In **d**, **e**, **k** and **m**, *n* = 3 biological replicates, the mean ± s.d. is shown, and the *P* values were calculated with the two-tailed Student’s *t*-test. Source data

**Extended Data Fig. 6:**
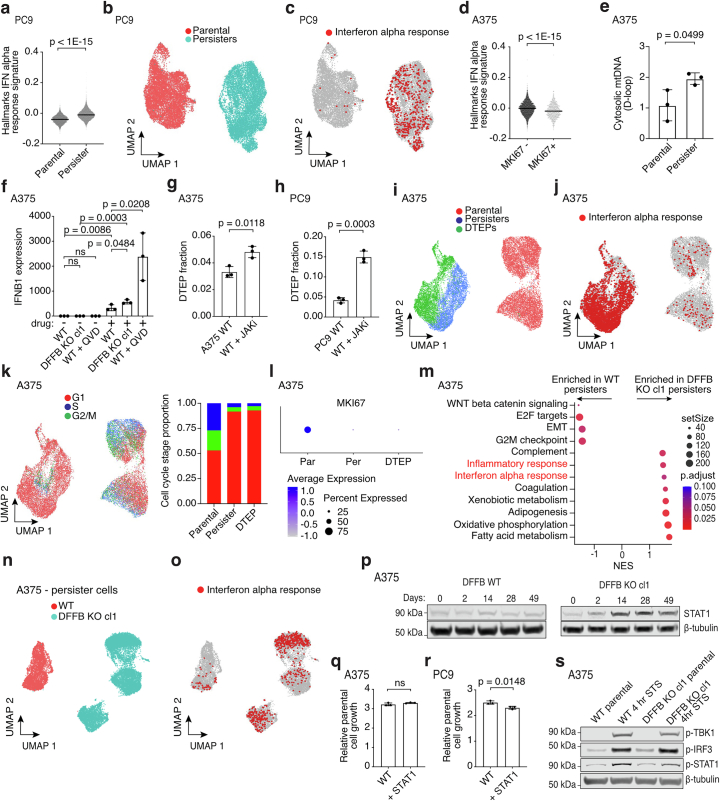
Increased STAT1 and IFN-stimulated genes restrict persister cell regrowth. **a**, ScRNAseq Hallmarks IFN alpha response gene set signature scores in PC9 cells. P values calculated with Mann-Whitney test. **b-c**, ScRNAseq UMAP visualizations of PC9 parental cells and persister cells derived from 2.5 μM erlotinib for 14 days (**b**) and cells with enriched Hallmarks IFN alpha response gene set highlighted in red (**c**). **d**, Hallmarks IFN alpha response gene set signature scores in A375 cycling (MKI67 + ) versus noncycling (MKI67-) DTEP cells. P value calculated with two-sided Mann-Whitney test. **e**, A375 cells analyzed for cytosolic mitochondrial DNA. **f**, IFNB1 mRNA expression in A375 cells treated with 1 μM dabrafenib and 100 nM trametinib for 6 days with and without 10 μM caspase inhibitor QVD treatment. cl, clone. **g**, DTEP formation from A375 WT persister cells treated with 250 nM dabrafenib and 25 nM trametinib for 2 weeks followed by the addition of 1 μM JAK inhibitor ruxolitinib for 5 weeks. **h**, DTEP formation from PC9 persister cells co-treated with 300 nM osimertinib and 5 μM ruxolitinib for 5 weeks. **i-k**, ScRNAseq UMAP visualizations of A375 parental cells, persister cells derived from treatment with 250 nM dabrafenib and 25 nM trametinib for 14 days, and DTEP cells treated for 9 weeks (**i**), cells with enriched Hallmarks IFN alpha response gene set highlighted in red (**j**), and cells colored by cell cycle stage (**k**). **l**, ScRNAseq MKI67 (Ki-67) expression in A375 parental (Par), persister (Per), and DTEP cells. **m**, Bulk RNAseq enriched Hallmarks gene sets between A375 DFFB KO versus WT persister cells. Normalized enrichment score, NES. EMT, epithelial-to-mesenchymal transition. P values adjusted with Benjamini–Hochberg correction. **n-o**, scRNAseq UMAPs of A375 WT and DFFB KO persister cells (**n**) and cells with enriched Hallmarks IFN alpha response gene set highlighted in red (**o**)**. p**, A375 cells treated with 250 nM dabrafenib and 25 nM trametinib and assessed for STAT1 at multiple timepoints. **q-r**, A375 (**q**) and PC9 (**r**) parental cell proliferation with or without overexpressed STAT1. **s**, A375 cells treated with 1 μM staurosporine (STS) for 4 h analyzed for activation of IFN signaling upstream of ISG expression. **e-h**,**q-r**, *n* = 3 biological replicates; mean $\pm$ s.d. is shown; P values calculated with two-tailed Student’s t-test. ns, not significant. Source data

The RNA-seq analysis of wild-type persister and DTEP cells revealed significantly enriched signatures composed of interferon (IFN)-stimulated genes (ISGs) in both populations compared with parental cells, though ISGs were decreased in cycling DTEP cells (Fig. 4a, Extended Data Fig. 6a–d and Supplementary Table 2). Tumour cell-intrinsic IFN signalling can contribute to growth arrest^26^ and suppress disseminated cancer cell growth^27^ and therefore could limit persister cell regrowth. One potential source of persister cell intrinsic IFN is MOMP, which, in apoptotic cells, exposes mitochondrial nucleic acids to cytoplasmic pattern recognition receptors that induce type I IFN^28^. We found that persister cells, which exhibit MOMP (Fig. 1g,h), also have an increased exposure of mitochondrial nucleic acids to cytosol and upregulated IFNβ expression (Extended Data Fig. 6e,f). We therefore hypothesized that persister cell regrowth is limited by intrinsic IFN signalling. Although a minority of wild-type persister cells regrow into DTEP colonies, most wild-type persister cells do not (Fig. 3a–e and Extended Data Fig. 3i), and we found that the cotreatment of persister cells with JAK inhibitor ruxolitinib to block IFN signalling increased the proportion of persister cells which regrew into DTEP colonies (Extended Data Fig. 6g,h). Furthermore, DTEP colonies are predominantly constituted by quiescent cells with increased ISGs, indicating that ISGs remain growth-suppressive throughout treatment (Extended Data Fig. 6i–l).

Interestingly, we found that drug-treated DFFB-KO cells exhibit strongly increased IFN transcriptional signatures above the corresponding wild-type cells at all timepoints (Fig. 4a–c, Extended Data Fig. 6m–o and Supplementary Tables 6–9). We also observed a stronger rescue of DTEP colony formation in DFFB-KO than in wild-type DFFB persister cells upon treatment with JAK inhibitor (Fig. 4d,e and Extended Data Fig. 6g,h). Furthermore, STAT1, a central IFN pathway transcription factor which drives prolonged expression of ISGs and is itself an ISG^29,30^, is upregulated throughout drug treatment in DFFB LOF cells compared with wild-type cells in a type I IFN signalling-dependent manner (Fig. 4f–i and Extended Data Fig. 6p). Increased STAT1 is sufficient to block persister cell regrowth because STAT1 ectopic overexpression suppresses wild-type DTEP formation (Fig. 4j–m). By contrast, increased STAT1 has minimal effect on parental cell proliferation, suggesting that IFN signalling in the absence of drug stress is insufficient to block growth (Extended Data Fig. 6q,r). These observations demonstrate that during drug treatment, DFFB suppresses IFN signalling sufficiently to allow the regrowth of a minority of persister cells. Consequently, the DFFB-deficient persister cells experience highly increased IFN signalling which prevents regrowth.

We then explored how DFFB regulates IFN signalling. Though high levels of DFFB-induced DNA damage were recently reported to promote transient STING-mediated type I IFN production during direct ectopic DFFB activation or acute viral infection^31^, persister cells have lower levels of drug stress-mediated DFFB-induced DNA damage, and this is insufficient to drive DFFB-dependent IFN induction (Extended Data Fig. 6f,s). Type I IFN production is instead regulated independent of DFFB in persister cells by apoptotic caspases as shown by increased IFNβ expression upon QVD treatment (Extended Data Fig. 6f), consistent with prior reports in other contexts^32,33^. Given that ISGs are strongly increased in DFFB-KO persister cells, but expression of type I IFN is not, we hypothesized that DFFB transcriptionally regulates ISGs downstream of IFN signalling. Though DFFB is a nuclease which has some preference for inducing DNA breaks in specific genomic regions^34^, and DFFB-induced DNA damage can affect proximal gene expression^35^, we considered it unlikely that DFFB induces coordinated suppressive DNA breaks at the numerous ISG loci DFFB regulates in persister cells. Therefore, we instead searched for an ISG suppressing factor, which is activated by DFFB.

### DFFB activates stress response factor ATF3

**Fig. 5:**
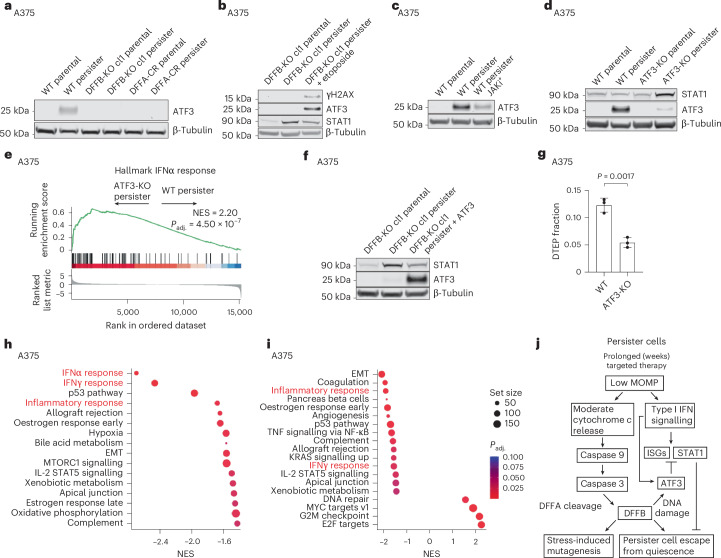
DFFB induces ATF3 to suppress ISGs and enable persister cell regrowth. **a**, ATF3 protein levels in A375 WT, DFFB-KO and DFFA-CR parental and persister cells. **b**, A375 DFFB-KO persister cells treated with or without 100 μM etoposide for 48 h and analysed for DNA damage, ATF3 and STAT1. **c**, A375 wild-type persister cells cotreated with 1 μM JAKi and analysed for ATF3 expression. **d**, Total STAT1 and ATF3 levels in A375 cells with CRISPR-mediated ATF3 depletion (pooled KO). **e**, Bulk RNA-seq gene set enrichment analysis of Hallmarks IFNα response gene set using differentially expressed genes between A375 ATF3-KO and wild-type persister cells. The *P* value was adjusted with the Benjamini–Hochberg correction. **f**, Total STAT1 and ATF3 protein levels in A375 DFFB-KO persister cells with ectopically expressed ATF3. **g**, DTEP colony formation in A375 wild-type and ATF3-depleted (pooled KO) cells. *n* = 3 biological replicates; mean ± s.d.; two-tailed Student’s *t*-test. **h**,**i**, Bulk RNA-seq gene set enrichment analysis of A375 ATF3-KO (**h**) and DFFB-KO (**i**) persister cells with versus without cotreatment with 20 μM AP1 inhibitor T-5224. The IFN-related gene sets are coloured red. *n* = 3 biological replicates. *P* values were adjusted with the Benjamini–Hochberg correction. **j**, Summary diagram. EMT, epithelial-to-mesenchymal transition. Source data

**Extended Data Fig. 7:**
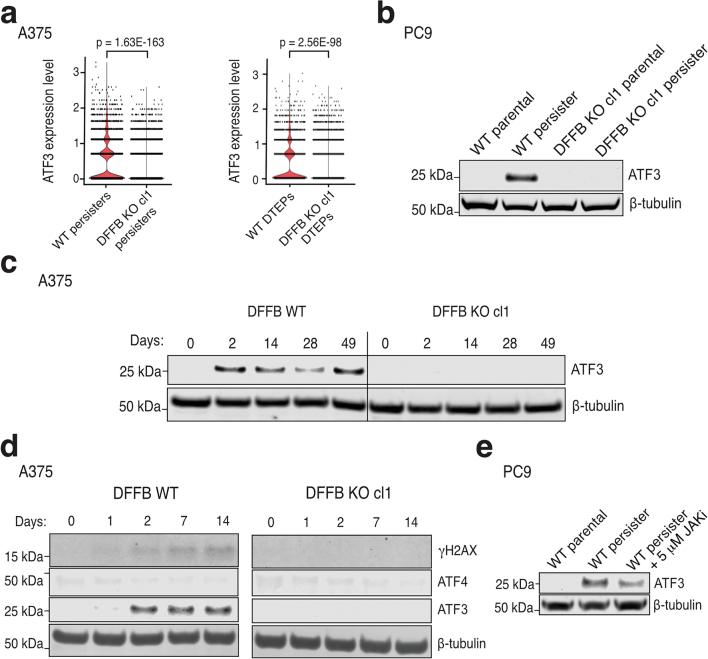
ATF3 induction in persister cells is dependent on DFFB. **a**, ATF3 expression levels between A375 WT and DFFB KO persister and DTEP cells measured with scRNAseq. P values calculated with the two-sided Wilcoxon Rank Sum test with Bonferroni correction. cl, clone. **b**, ATF3 expression in PC9 WT and DFFB KO cells treated with 500 nM osimertinib. **c**, A375 WT and DFFB KO cells treated with 250 nM dabrafenib and 25 nM trametinib analyzed for ATF3 at the indicated times. **d**, A375 WT and DFFB KO cells treated with 250 nM dabrafenib and 25 nM trametinib analyzed for γH2AX, ATF4, and ATF3 at the indicated times. **e**, PC9 persister cells derived from 500 nM osimertinib with or without 5 μM JAK inhibitor ruxolitinib and analyzed for ATF3 levels. Source data

We found that stress response factor ATF3 is specifically induced in drug-treated wild-type cells at both the persister and DTEP timepoints (Fig. 5a and Extended Data Fig. 7a–c). ATF3 has previously been shown to either negatively or positively regulate the expression of ISGs including STAT1 in other contexts^36–39^. Therefore, we considered ATF3 to be a candidate mediator of DFFB-induced ISG suppression. We first tested how ATF3 expression is induced in persister cells. ATF3 can be induced by a variety of stresses and signals including DNA damage^40^, IFN signalling^41^ and the integrated stress response (ISR)^42^. ATF3 is induced in wild-type persister cells which have DNA damage and is not induced in DFFB-KO persister cells which lack DNA damage (Extended Data Fig. 7d). Furthermore, ATF3 expression is rescued in DFFB-KO persister cells by treatment with topoisomerase inhibitor etoposide, which induces DNA breaks independent of DFFB^43^, thereby demonstrating that DNA damage drives ATF3 induction in persister cells (Fig. 5b). We also found that JAK inhibitor cotreatment reduced ATF3 levels in persister cells, indicating that IFN signalling also promotes ATF3 induction (Fig. 5c and Extended Data Fig. 7e).

**Extended Data Fig. 8:**
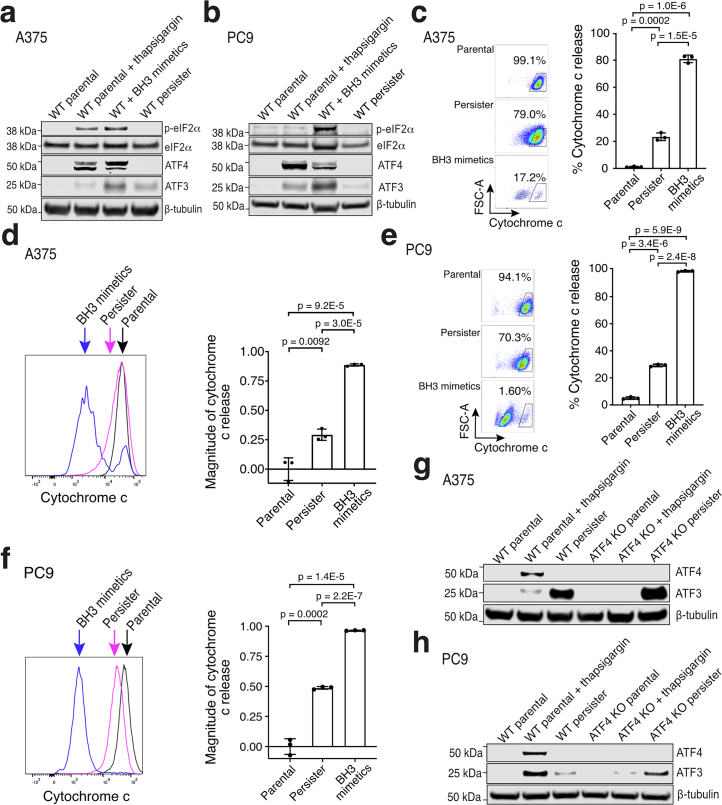
ATF3 is induced in persister cells independent of the integrated stress response. **a**, A375 WT cells treated with ER stress inducer 1 μM thapsigargin for 4 h, BH3 mimetics 5 μM ABT-737 and 10 μM S63845 for 2.5 h followed by 24 h recovery, and persister cells analyzed for ISR genes (phosphorylated (p) eIF2α, total eIF2α, ATF4) and ATF3. **b**, PC9 WT cells treated with 1 μM thapsigargin, BH3 mimetics 1.5 μM ABT-737 and 3 μM S63845 for 4 h followed by 2 h recovery, and persister cells analyzed for ISR genes and ATF3. **c-f**, Flow cytometry analysis of cytochrome C release in A375 (**c,d)** and PC9 (**e**,**f**) parental, persister, and BH3 mimetic-treated cells. **d** and **f**, Fractional loss of cytochrome C (geometric means) are plotted on the right graphs. *n* = 3 biological replicates; mean $\pm$ s.d. is shown; P values calculated with two-tailed Student’s t-test. **g,h**, A375 (**g**) and PC9 (**h**) WT and ATF4 CRISPR-depleted (pooled KO) persister cells were analyzed for ATF3 levels. Source data

The ISR is activated by a variety of stresses resulting in the phosphorylation of eIF2α and the induction of ATF4, which drives the expression of target genes including ATF3^44^. Recently, the ISR was shown to also be induced via haem-regulated inhibitor kinase activation as a result of MOMP and cytochrome *c* release in cancer cells which recover from transient treatment with cytotoxic concentrations of BH3 mimetics, resulting in cells with persister cell features and metastatic potential^9^. We also observed that transient treatment with BH3 mimetics induces the ISR and induction of ATF3 in recovered A375 and PC9 cells (Extended Data Fig. 8a,b). However, we failed to detect phosphorylated eIF2α or increased ATF4 at any timepoint during targeted therapy treatment, including in persister cells (Extended Data Figs. 7d and 8a,b). An explanation for this distinction is that cytotoxic BH3 mimetic-treated cells exhibit much higher mitochondrial cytochrome *c* release compared with targeted therapy-treated persister cells, suggesting that a minimum threshold of cytoplasmic cytochrome *c* is required to activate the ISR (Extended Data Fig. 8c–f and Supplementary Fig. 6). Furthermore, whereas ATF3 induction is dependent on ATF4 during the ISR, we found that, in persister cells, ATF3 is induced independently of ATF4 because ATF4-KO persister cells robustly induce ATF3 (Extended Data Fig. 8g,h). Therefore, DFFB-dependent ATF3 induction in persister cells is mediated by DNA damage and IFN signalling, not the ISR.

### ATF3 suppresses ISG expression to promote persister cell regrowth

**Extended Data Fig. 9:**
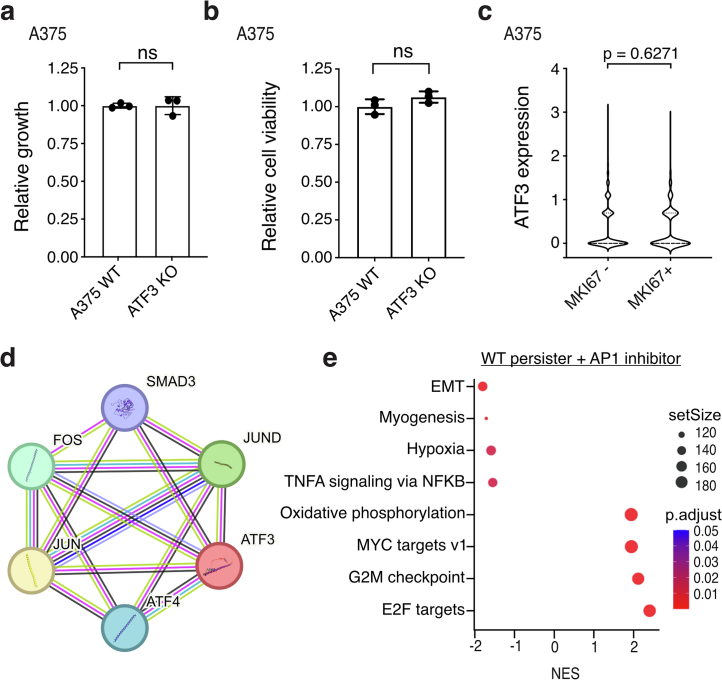
Additional ATF3 and AP1 data. **a**,**b**, A375 ATF3-depleted (pooled KO) cells assessed for parental cell proliferation (**a**) and persister cell formation (**b**). *n* = 3 biological replicates; mean $\pm$ s.d. is shown; P values calculated with two-tailed Student’s t-test. ns, not significant. **c**, A375 WT DTEP cycling (MKI67 + ) and noncycling (MKI67-) cell ATF3 scRNAseq expression. P values calculated with the two-sided Wilcoxon Rank Sum test. **d**, STRING analysis of ATF3 interactions. **e**, Bulk RNAseq gene set enrichment analysis of A375 WT persister cells versus persister cells co-treated with 20 μM AP1 inhibitor T-5224. *n* = 3 biological replicates. EMT, epithelial-to-mesenchymal transition. Source data

We next tested whether ATF3 functionally suppresses ISGs in persister cells to allow regrowth and found that CRISPR-mediated ATF3 depletion resulted in increased STAT1 and IFN-related gene sets (Fig. 5d,e and Supplementary Tables 10 and 11). Furthermore, in DFFB-KO persister cells, which fail to induce ATF3 (Fig. 5a), the ectopic re-expression of ATF3 decreased STAT1 levels (Fig. 5f). Similarly, the rescue of ATF3 expression in DFFB-KO persister cells by etoposide treatment also suppressed STAT1 (Fig. 5b). Furthermore, similar to DFFB LOF cells, ATF3-depleted persister cells are hindered in their ability to regrow into DTEP colonies (Fig. 5g). Likewise similar to DFFB LOF cells (Extended Data Fig. 4c–p), the decreased DTEP colony formation of ATF3-depleted cells was not due to any defect in proliferation or persister cell viability (Extended Data Fig. 9a,b). ATF3 also remained highly expressed in wild-type DFFB but not in knockout cells at the timepoint wild-type DTEP colonies form, consistent with a continuous role for ATF3 in suppressing ISGs throughout DTEP colony formation (Extended Data Figs. 7c and 9c).

We next sought to determine how ATF3 suppresses ISG expression in persister cells. ATF3 binds and antagonizes activator protein 1 (AP1) transcription factor complex proteins^45^ (Extended Data Fig. 9d), and AP1 was recently reported to drive memory inflammatory gene expression, including ISGs in epidermal stem cells^46^. We therefore tested whether ATF3 suppression of ISGs in persister cells occurs as a result of negative regulation of AP1 transcription factor activity. Consistent with this, we observed that the cotreatment of DFFB-KO and ATF3-depleted persister cells, which are each deficient in ATF3 and have increased ISGs (Figs. 4a–c and 5a,d,e and Extended Data Figs. 6m–o and 7a–c), with AP1 inhibitor T-5224 decreased expression of IFN-related gene sets (Fig. 5h,i and Supplementary Tables 12–15). By contrast, AP1 inhibitor treatment has no effect on IFN-related gene sets in wild-type persister cells as expected for cells with intact ATF3-mediated AP1 inhibition (Extended Data Fig. 9e and Supplementary Tables 16 and 17). These data support ATF3-mediated AP1 inhibition as a mechanism by which DFFB suppresses ISGs in persister cells.

### On-treatment patient tumours exhibit features of IFN-enforced persister cell growth arrest

**Extended Data Fig. 10:**
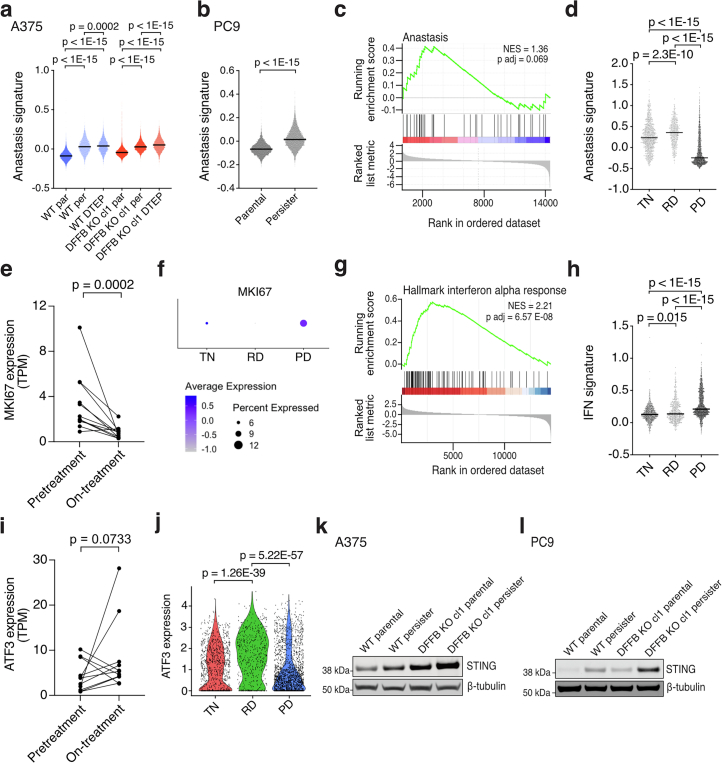
Analysis of persister cell signatures within patient tumours. **a**,**b**, ScRNAseq anastasis gene set signature scores in A375 parental, persister, and DTEP timepoint DFFB WT and KO cells (**a**) and PC9 parental and persister cells (**b**). Anastasis remains elevated at the DTEP timepoint, indicating that DTEP cells remain drug stressed. cl, clone. **c**, Anastasis gene set expression analyzed in on-treatment versus pretreatment patient melanoma tumours treated with BRAF + /- MEK targeted therapy. Normalized enrichment score, NES. P value calculated with a two-sided permutation test with family-wise error rate correction. **d**, Anastasis gene set signature scores in treatment naïve (TN), residual disease (RD), and progressive disease (PD) patient non-small cell lung cancer tumours treated with EGFR targeted therapy. **e**, MKI67 (Ki-67) expression in pretreatment and on-treatment melanoma patient tumours. **f**, Ki-67 expression in each lung cancer treatment response stage. **g-h**, Hallmarks IFN alpha response gene set expression analyzed in on-treatment versus pretreatment patient melanoma (**g**) and lung cancer tumour treatment response stages (**h**). **g**, P value adjusted with Benjamini–Hochberg correction. **i**, ATF3 expression in pretreatment and on-treatment patient melanoma tumours. **j**, ATF3 expression in each lung cancer treatment response stage. P value calculated with the two-sided Wilcoxon Rank Sum test with Bonferroni correction. **k,l**, A375 (**k**) and PC9 (**l**) WT and DFFB KO parental and persister cells analyzed for STING expression. **a,b,d**,**h**, P values calculated with two-sided Mann-Whitney test. **d,h**, *n* = 1,073 (TN), 572 (RD), and 2,109 (PD) cells analyzed from 15 (TN), 12 (RD), and 17 (PD) patients. **e**,**i**, *n* = 11 patients. P values calculated with two-sided paired ratio t-test. Source data

To assess evidence for our findings in patients, we explored datasets from tumours from patients with melanoma and lung cancer which were analysed by RNA-seq before and during targeted therapy treatment^47,48^. We first investigated the anastasis signature which marks drug-stressed persister and DTEP cells (Fig. 1b–f and Extended Data Fig. 10a,b) and found that it is increased in both on-treatment melanoma samples and residual disease lung cancer samples collected during treatment (Extended Data Fig. 10c,d). By contrast, the progressive disease lung cancer samples collected at later timepoints are markedly devoid of the anastasis signature, suggesting that progressive disease tumours have acquired resistance and do not experience drug stress (Extended Data Fig. 10d). Indeed, many of the progressive disease tumours were reported to harbour mutations and non-genetic mechanisms which can cause resistance^49^.

Similar to persister and DTEP cells (Extended Data Fig. 6l), both the on-treatment melanoma and residual disease lung cancer samples have decreased expression of proliferation marker Ki-67 (Extended Data Fig. 10e,f), whereas progressive disease tumours have high levels of Ki-67 (Extended Data Fig. 10f). On-treatment melanoma and residual disease lung cancer samples are also enriched for ATF3 and exhibit moderately increased expression of ISGs (Extended Data Fig. 10g–j) similar to wild-type DFFB persister and DTEP cells (Figs. 4a and 5a, Extended Data Fig. 6a–c,i,j and 7a–c). By contrast, consistent with a lack of drug stress, progressive disease tumours fail to induce ATF3 (Extended Data Fig. 10j) and exhibit even further increased ISGs (Extended Data Fig. 10h). Without drug stress to induce tumour cell-intrinsic IFN production, the source of IFN in progressive disease may be primarily from immune infiltrate^49^. Despite high ISGs, progressive disease tumours are proliferative, suggesting that in the absence of drug stress, IFN alone is insufficient to cause tumour growth arrest. Indeed, we also found that increased STAT1 has a minimal effect on the proliferation of unstressed parental cells (Extended Data Fig. 6q,r) yet blocks drug-stressed persister cell regrowth (Fig. 4j–m). Together, these analyses demonstrate that multiple core features of wild-type DFFB persister and DTEP cells are present within drug-stressed residual tumours including anastasis, ATF3 and IFN regulation, but that untreated or fully resistant tumours which avoid drug stress lack these features.

## Discussion

The adaptive mechanisms utilized by cancer cells to survive drug treatment and develop resistance are poorly understood. Although genetic resistance mutations often drive long term acquired drug resistance, the earliest events which enable residual cancer cells to regrow may be non-genetic^49^. By exploring the consequences of sublethal apoptosis in persister cells, we uncovered a regulatory mechanism in which apoptotic DNase DFFB orchestrates the transcriptional repression of ISGs, enabling escape from IFN-enforced growth arrest and facilitating initial persister cell regrowth (Fig. 5j). Cancer cells deficient in DFFB are notably unable to regrow during targeted therapy treatment. DFFB also promotes mutagenesis in persister cells, which may contribute to cancer stress-induced mutagenesis^3,4,25^. These findings are consistent with clinical observations that increased apoptosis signal in regressed tumours during drug treatment predicts worse outcome^50^.

We found DFFB-mediated DNA damage induces ATF3, which accumulates during drug treatment and functions as part of IFN negative feedback machinery that limits persister cell ISG expression. It was recently shown that cancer cells which survive brief treatment with cytotoxic concentrations of BH3 mimetics induce MOMP and cytochrome *c* release, which activates the ISR including ATF4 and its target gene ATF3^9^. We did not observe ISR activation or ATF4-mediated ATF3 expression in targeted therapy-treated persister cells, which exhibit lower MOMP and cytochrome *c* release compared with cells which survive brief cytotoxic BH3 mimetic treatment. Based on these observations, we propose that there are distinct outcomes for cancer cells depending on the level and duration of MOMP they experience. Cancer cells which survive transient high levels of MOMP can activate the ISR in a caspase-independent manner to form persister cells and gain metastatic potential^9^, whereas targeted therapy-treated persister cells experience chronic moderate levels of MOMP and instead undergo caspase-dependent DFFB-mediated ISG suppression which promotes regrowth without affecting the persister cell formation. Given these disparate molecular mechanisms and phenotypic consequences of apoptotic stress, it seems likely that there are additional features of sublethal death signalling that remain to be discovered.

Whereas DFFB-deficient persister cells are almost completely unable to regrow owing to high levels of IFN signalling, most wild-type persister cells also remain growth arrested, and only a small minority regrow into DTEP colonies. It is unclear why a subset of wild-type persister cells regrow and most do not. One possibility is heterogeneity in DFFB and IFN signalling levels. Indeed, persister cells with higher levels of caspase activity have higher levels of DFFB-induced DNA damage (Extended Data Fig. 2j), and there is also some heterogeneity in persister cell ISG expression such that DTEP cells that are proliferating have lower levels of ISGs (Fig. 4a–c and Extended Data Fig. 6a–d,i,j,n,o). However, given that ATF3 levels are similar among cycling and non-cycling DTEP cells (Extended Data Fig. 9c), additional adaptations probably contribute to DTEP cell cycling which remain to be determined. Beyond IFN suppression, there are other hurdles that persister cells also probably need to overcome to escape quiescence, including the re-establishment of mitogenic signalling and metabolic processes^51^. Therefore, although DFFB is a critical factor to enable DTEP formation, a full understanding of the transition from quiescent persister cell into cycling DTEP cell will require additional study.

DFFB is a non-essential gene in mice and a potentially druggable enzyme^52–54^. Despite being a member of the apoptotic pathway, DFFB is not required for the execution of cell death^55^ as DFFB-KO cells respond similarly to drug as wild-type cells (Extended Data Fig. 4) and display both increased cleaved caspase 3 (Extended Data Fig. 4q) and anastasis signatures (Extended Data Fig. 10a). In addition to our findings that DFFB-deficient persister cells and residual tumours are unable to regrow during targeted therapy treatment, DFFB-deficient tumours are also sensitized to radiation^34^. DFFB inhibition may also promote tumour immunity through the upregulation of immune-stimulating ISGs such as STING^56^ specifically within drug-stressed tumour cells (Extended Data Fig. 10k,l). Indeed, because DFFB is inactive and bound to DFFA in unstressed cells, it may be possible to achieve DFFB inhibition specifically within tumour cells under targeted therapy or other stress. Therefore, DFFB is a unique adaptation factor leveraged by cancer cells to adapt to drug stress.

## Methods

### Ethical statement

The University of California San Francisco (UCSF) Institutional Animal Care and Use Committee approved the mouse xenograft studies which were performed at the UCSF Preclinical Therapeutics Core in protocol no. AN179937.

### Cell lines and culture

A375 (CRL-1619) and BT474 (HTB-20) cells were purchased from the American Type Culture Collection. PC9 cells were provided by the Altschuler and Wu Lab at UCSF. A375 cells were cultured in Dulbecco’s modified Eagle medium (DMEM; Thermo Fisher Scientific) supplemented with 10% foetal bovine serum (FBS) and 1% antimycotic-antibiotic (Thermo Fisher Scientific). PC9 cells were cultured in Roswell Park Memorial Institute 1640 medium (Thermo Fisher Scientific) supplemented with 5% FBS and 1% antimycotic-antibiotic. BT474 cells were cultured in Roswell Park Memorial Institute 1640 medium supplemented with 10% FBS and 1% antimycotic-antibiotic. All cell lines were maintained in 5% CO_2_ atmosphere at 37 °C. Cell lines were split with 0.25% Trypsin-EDTA (Thermo Fisher Scientific). Cell line identities were confirmed with short tandem repeat profiling at the University of California Berkeley Cell Culture Facility. All cell lines regularly tested negative for mycoplasma throughout these investigations using the Lonza Mycoalert Mycoplasma Detection Kit.

### Chemical and antibody sources for cell treatments

Dabrafenib (S2807), trametinib (S2673), erlotinib (S7786), osimertinib (S7297), lapatinib (S2111), staurosporine (S1421), puromycin (S9631), doxycycline hyclate (S4163) and quinoline-Val-Asp-difluorophenoxymethylketone (QVD) (S7311) were purchased from Selleck Chemicals. Etoposide (E55500) was purchased from Research Products International. JC-1 dye (T3168) and digitonin (AC407565000) were purchased from Thermo Fisher Scientific. Ghost Dyes Violet 510 (13-0870-T100) and Red 780 (13-0865-T100) were purchased from Tonbo Biosciences. Carbonyl cyanide *m*-chlorophenyl hydrazone was purchased from Abcam (ab141229). BioTracker NucView 530 Red Caspase 3 Dye (PBS) (SCT105) and cytochalasin B (C2743) were purchased from Sigma-Aldrich. JAK inhibitor ruxolitinib (HY-50856), TBK1 inhibitor MRT67307 (HY-13018), thapsigargin (HY-13433), ABT-737 (HY-50907), S63845 (HY-100741) and T-5224 (HY-12270) were purchased from MedChemExpress. Anti-IFNα/β receptor 1 antibody (ab10739) was purchased from Abcam.

### Drug and chemical treatments

Previously described protocols were used^6,7^. In brief, A375 cells were treated with 250 nM dabrafenib and 25 nM trametinib for 14 days; PC9 cells were treated with 2.5 μM erlotinib for 10 days, or with 300 nM or 500 nM osimertinib for 9 days; and BT474 cells were treated with 500 nM or 2 μM lapatinib for 10 days to generate persister cells, unless otherwise indicated. A375 cells were treated with 250 nM dabrafenib and 25 nM trametinib for 7 weeks for DTEP cell analyses, unless otherwise indicated. The prederived persister cells were assayed while still in drug, unless otherwise noted. In experiments utilizing caspase inhibitor QVD, 10 μM QVD was added together with the respective targeted therapy treatment for the duration of the experiment. Media and drug were replenished every 3–4 days. For the etoposide treatment of DFFB-KO persister cells, 100 μM etoposide was added to preformed persister cells for 48 h before 24 h of recovery without any drugs before protein collection. The control DFFB-KO persister cells for this experiment (Fig. 4b) similarly received a 24-h recovery without any drugs before protein collection. For the thapsigargin treatments, cells were treated with 1 μM thapsigargin for 4 h. For the BH3 mimetic treatments, A375 cells were treated with 5 μM ABT-737 and 10 μM S63845 for 2.5 h then allowed to recover in media without drugs for 24 h. PC9 cells were treated with 1.5 μM ABT-737 and 3 μM S63845 for 3 h then allowed to recover in normal media without drugs for 2 h.

### DTEP colony formation

Cells were plated at low starting densities to avoid the outgrowth of overlapping DTEP colonies. Drug media were refreshed every 3–4 days. At 7 weeks in drug for A375 cells, 5 weeks in drug for PC9 cells and 11 weeks in drug for BT474 cells, plates were fixed with 90% methanol and stained with crystal violet. The observer was blinded to the treatment conditions of the plates before colony counting by microscopy. Colonies with greater than 25 cells were considered DTEP colonies. To calculate the fraction of cells that regrew into DTEP colonies (‘DTEP fraction’), the number of DTEP colonies per plate was divided by the sum of the number of isolated single cells plus colonies of any size.

### Microscopy

Brightfield photos were captured with the EVOS XL Core microscope using the 4× LPlan PH2 objective.

### Cell viability

Cell viability was assessed with CellTiter Glo (CTG) 2.0 Cell Viability assay (Promega, G7571). Luminescence was read with a Molecular Devices SpectraMax iD3 plate reader with SoftMax Pro 7 software.

### Single-cell RNA-seq

A375 cells were cultured with 250 nM dabrafenib and 25 nM trametinib, and the PC9 cells were treated with 2.5 μM erlotinib for 2 weeks to establish persister cells. For the DTEP timepoint, A375 wild-type and DFFB-KO cells were cultured with 250 nM dabrafenib and 25 nM trametinib for 9 weeks. For all conditions, an untreated parental control was cultured alongside the drug-treated conditions. Cells were then lifted with trypsin and libraries were generated using the 10x Chromium Single Cell 3′ v3 kit (10x Genomics). Quality control of the libraries was conducted with the High Sensitivity D1000 ScreenTape kit with the Agilent TapeStation and then sequenced using a NovaSeq S4 flowcell on an Illumina NovaSeq 6000 sequencer at the UCSD Institute for Genomic Medicine.

### Single-cell RNA-seq data processing and mapping

The fastq files were aligned to the human ‘refdata-cellranger-GRCh38-3.0.0’ genome with 10x Genomics Cell Ranger 3.1.0^57^. The ‘filtered_feature_bc_matrix’ was used in the Seurat R package version 4.0.3^58–60^. Cells containing greater than 1,000 and less then 7,500 features, and with less than 20% mitochondrial reads, were included in downstream analyses. A cell cycle score was calculated using the default Seurat method^61^ and was used to regress cell cycle during normalization and scaling with the ‘SCTransform’ command. To generate uniform manifold approximation and projection (UMAP) plots, the commands ‘RunPCA,’ ‘RunUMAP,’ ‘FindNeighbors,’ and ‘FindClusters’ were performed with default settings, with 30 dimensions used for ‘RunUMAP’ and ‘FindNeighbors’. In the ‘FindClusters’ command, the resolution for A375 and PC9 was set to 1.0 and 0.15, respectfully. For cluster analyses within the persister cell populations only, the resolution was set to 0.2 for A375 and 0.05 for PC9. The Seurat command ‘FindMarkers’ was used without thresholds for fold change or percentage of gene-expressing cells to calculate differentially expressed genes.

### Single-cell RNA-seq persister cell gene signature analysis

A gene set enrichment analysis was conducted with the ClusterProfiler R package (version 3.18.0)^62^. The minimum gene set size for analysis was set to five, and the Benjamini–Hochberg method was used for false discovery rate correction. The signature scoring was performed using the Seurat command ‘AddModuleScore’. To display the enriched Hallmarks IFNα response gene set on a UMAP, AUCell package version 1.12.0 was used to calculate gene set scores per cell^63^.

### JC-1 mitochondrial membrane potential assay

A375 persister cells were derived and untreated parental cells were cultured alongside. Cells were then lifted with trypsin for JC-1 staining. As a positive control, the cells were treated with 50 µM carbonyl cyanide *m*-chlorophenyl hydrazone for 5 min. Cells were stained with 1.5 µM JC-1 for 30 min away from light at 37 °C. The cells were then stained with Ghost Dye Red 780 (Tonbo Biosciences, 13-0865-T100) diluted 1:1,000 in phosphate-buffered saline (PBS; Gibco, 10010023) for 15 min away from light at room temperature. The cells were washed in PBS and analysed on a BD FACSCanto RUO flow cytometer. At least 30,000 live cell events were collected per sample. Flow cytometry results were analysed using FlowJo Software (BD Life Sciences) version 10.7.1. See Supplementary Fig. 2 for the gating strategy.

### Immunoblotting

Persister or DTEP cells were derived with drug treatment in 10- or 15-cm plates. Cells were then washed with PBS and lysed using radioimmunoprecipitation assay buffer (Thermo Fisher Scientific, 89900) supplemented with phosphatase inhibitor (Thermo Fisher Scientific, 78420) and protease inhibitor (Thermo Fisher Scientific, 78430). Lysates were centrifuged at 14,000*g* at 4 °C for 15 min, and the protein concentration of the supernatant was determined using the Pierce BCA Protein Assay Kit (Thermo Fisher Scientific, 23225). The lysates were mixed with sample buffer (Thermo Fisher Scientific, NP0007) and denatured at 70 °C for 10 min. Samples were separated by SDS–PAGE (Bolt 4–12% Bis–Tris Gel, Life Technologies, NW04120BOX), run with Chameleon Duo Prestained Protein Ladder (LICOR, 928-60000) and transferred to a nitrocellulose membrane using the iBLOT 2 Dry Blotting System (Life Technologies, IB21001). Membranes were blocked with 5% bovine serum albumin for 1 h at room temperature and then incubated with primary antibody at 4 °C overnight. LICOR secondary antibodies were then incubated with the membrane for 1 h at room temperature, and the membranes were imaged using the LICOR Odyssey Imaging System and Image Studio version 5.2. Antibody commercial sources are provided in Supplementary Table 18.

### DNA damage, cleaved caspase 3 and cytochrome *c* flow cytometry

A375 persister cells were derived, and untreated parental cells were cultured along with them. For the γH2AX experiments, A375 persister cells treated with 10 µM QVD for the duration of drug treatment were also prepared.Cells were then trypsinized and collected for staining. Cells were stained with viability dye Ghost Dye Red 510 or 780 for 15 min away from light at room temperature. For cytochrome *c* staining, cells were incubated in 50 µg ml^−1^ digitonin in PBS for 10 min on ice^64^. Cells were fixed with 4% paraformaldehyde for 10 min at room temperature. For γH2AX and cleaved caspase 3 staining, the cells were permeabilized with 0.3% Triton X-100 in PBS for 10 min at room temperature. Cells were stained for proteins of interest using primary conjugated antibodies at manufacturer-recommended dilutions in PBS for 30 min to 1 h at room temperature. Cells were washed in PBS and analysed on a BD FACSCanto RUO flow cytometer. At least 30,000 live cell events were collected per sample. Flow cytometry data were analysed using FlowJo Software (BD Life Sciences) version 10.7.1. See Supplementary Figs. 1–6 for the gating strategies.

### Caspase 3/7 activity reporter assay

Caspase activity was measured using NucView 530 Caspase 3 Substrate (Biotium, 10408). The positive control cells were derived from 4-h treatment with 1 µM staurosporine. The cells were collected by trypsinization, washed in PBS and incubated with Ghost Dye Violet 510 cell viability dye following the manufacturer’s instructions. Cells were washed with PBS plus 1% FBS and subsequently incubated with 5 µM NucView 530 Caspase 3 Substrate for 30 min in PBS plus 1% FBS and analysed by flow cytometry using a BD FACSAria II sorter. See Supplementary Fig. 4 for the gating strategy. Sorted live cells were collected in PBS plus 10% FBS and used for downstream viability assays. For testing caspase 3/7 activity-positive persister cell regrowth, the cells were sorted and plated in drug-free media; 24 h later, CTG was performed to measure cell viability. To assess regrowth ability, drug-free media were refreshed on day 3, and CTG was performed on day 6. DNA damage levels for A375 persister cells with basal and medium caspase activity were measured by sorting cells and extracting protein for western blot analysis.

### CRISPR editing

Caspase 9-KO cells were generated with Santa Cruz caspase 9 CRISPR plasmids (h) (sc-400257-KO-2), ATF3-KO cells with Santa Cruz ATF3 CRISPR plasmids (h) (sc-416577) and ATF4-KO cells with Santa Cruz ATF4 CRISPR plasmids (h2) (sc-400155-KO-2) following manufacturer’s protocols. In brief, caspase 9 CRISPR plasmid was cotransfected with caspase 9 HDR plasmid (h2) (sc-400257-HDR-2); ATF3 CRISPR plasmid was cotransfected with ATF3 HDR plasmid (h) (sc-416577-HDR); and ATF4 CRISPR plasmid was cotransfected with ATF4 HDR plasmid (h2) (sc-400155-HDR-2) using UltraCruz Transfection Reagent (sc-395739) into A375 or PC9 cells. The cells were then selected with puromycin; the expanded and edited cell pools were analysed for depletion of target protein via western blot. The caspase 9-depleted cells also underwent clonal selection to identify cells with complete knockout, and the depleted cells (pooled KO) or the clonal complete-KO clones were utilized in experiments as described. The ATF3- and ATF4-depleted (pooled KO) cell pools were used without further clonal selection. The DFFB CRISPR editing was performed at the UCSF Cell and Genome Engineering Core. Human DFFB single guide RNA (sgRNA) g1 was previously reported^14^. Additional guide RNAs (gRNAs) targeting the first (g4) or second (g2r) exon of human DFFB were designed and cloned into the eSpCas9(1.1) vector from the Zhang laboratory at MIT (Addgene plasmid 71814; Research Resource Identifier: Addgene_71814). A375 DFFB-KO clone no. 1 was constructed with sgRNA g1; A375 DFFB-KO clone no. 2 was constructed with g4; PC9 DFFB-KO clones were both constructed with g2r; and BT474 DFFB-KO clones were both constructed with g4. See Supplementary Table 19 for the guide sequences.

Following sequence verification, the guide constructs were introduced individually either by transfection or electroporation. For transfection, the guide constructs were cotransfected with a vector expressing mCherry and puromycin resistance using Lipofectamine 3000. Transfected cells were selected with puromycin. For electroporation, the cells were electroporated with the CRISPR–Cas9 RNP gRNAs and high-fidelity Cas9p (Integrated DNA Technologies) using the Lonza 4D Nucleofector kit SF with programme FF-120. After the cell expansion, genomic DNA was extracted from a portion of the cell pool (Nucleospin Blood gDNA extraction kit, Takara Bio), and the target sites were amplified (Phusion DNA polymerase, NEB) and sequenced using the primers indicated in Supplementary Table 19 (Sanger sequencing by GeneWiz; primers and sgRNA cloning oligonucleotides from Integrated DNA Technologies). CRISPR–Cas9 efficiency was determined by TIDE analysis (tide.deskgen.com). Clones which showed only frameshift-causing indels in all alleles were further analysed by TOPO cloning (Life Technologies) and Sanger sequencing and with the ICE analysis tool (Synthego). Clones with a DFFB-KO genotype were subsequently confirmed to lack DFFB protein expression by western blot (Extended Data Fig. 3d–f).

### Lentivirus production

*Escherichia coli* cultures with lentiviral vectors were grown on LB Agar Ampicillin-100 plates (Sigma-Aldrich, L5667). A colony was picked and grown overnight in LB-amp and plasmid DNA was isolated using the Qiagen HiSpeed Plasmid Midi Kit (Qiagen, 12643) and quantified with a Nanodrop. HEK293T cells were transfected with packaging plasmids psPAX2 (Addgene, 12260) and pMD2G (Addgene, 12259) in Opti-MEM (Thermo Fisher Scientific, 31985062) and polyethylenimine (Sigma-Aldrich, 764604). Virus-containing supernatant was collected, centrifuged and then filtered with a 0.45-μm filter (Sigma-Aldrich, SE1M003M00).

### Ectopic gene expression

Doxycycline-inducible DFFB expression vectors, STAT1 overexpression vector and ATF3 vector were obtained from VectorBuilder. The cleavage-resistant DFFA (DFFA-CR) short isoform construct was provided by the Elledge lab^21^. DFFB expression was driven by the third generation tetracycline-responsive element promoter; STAT1 expression was driven by the ubiquitin C promoter; and ATF3 expression was driven by the cytomegalovirus immediate early enhancer/promoter. All the vectors contained a puromycin resistance gene driven by the phosphoglycerate kinase 1 promoter. The DFFA-CR construct was transduced into A375 wild-type cells, STAT1 overexpression construct into A375 wild-type and PC9 wild-type cells and wild-type DFFB (NM_004402.4), nuclease-deficient DFFB (H260N)^22^ or human ATF3 into A375 DFFB-KO cells. Cells were then selected with puromycin. A single-cell clone confirmed for DFFA-CR expression was isolated and expanded for experiments. For doxycycline-inducible DFFB, the cells were seeded and 1.0 μg ml^−1^ doxycycline was added the following day. Media with doxycycline were refreshed every 3–4 days for the duration of the experiment.

### Whole-exome sequencing

A single A375 or PC9 wild-type or DFFB-KO cell was used to begin the experiments. Following initial expansion for 3 weeks, a portion of the cell population was frozen as the reference and the remaining cells were utilized for drug treatments. The respective drugs were added the following day, and drug media were refreshed every 3–4 days. Following 7 weeks of drug treatment for A375 and 10 weeks of treatment for PC9, each plate was allowed to regrow without drug for 10 days, the cells were lifted with trypsin and multiple individual cells were isolated through single-cell bottlenecks in 384-well plate wells. Single cells were expanded for 5–6 weeks in drug-free media to accumulate adequate genomic DNA content for sequencing. Frozen cell pellets had DNA extracted and libraries prepared for whole-exome sequencing (Agilent SureSelect V6 58 M) or whole-genome sequencing (A375 pretreatment reference sample, NEB Ultra II) at Novogene and were sequenced on an Illumina NovaSeq 6000 PE150.

### Single nucleotide variant analysis

Each sequenced sample was analysed according to the GATK best practices^65–67^. GATK version 4.2.4.0 pipelines were used. Fastq files were mapped to the hg38 reference genome provided in the GATK shared resource bucket with BWA^68^. Duplicate reads were marked with Picard, and the base scores were recalibrated with the GATK tool ‘BaseRecalibrator’. Mutect2 was used to call mutations between the drug-treated sample and the respective reference sample. Sequencing errors and contamination were estimated with ‘GetPileUpSummaries’, ‘CalculateContamination’ and ‘FilterMutectCalls’. Variants were annotated with ‘Funcotator’. Low frequency (<0.4) mutations were excluded from the analysis. Output files were analysed with the Maftools R Bioconductor package version 2.6.05^69^.

### In vivo tumour acquired resistance

Sample size was determined on the basis of prior experience with this model^7^. A375 cells were cultured in DMEM with 10% FBS before implantation. Cells were suspended in a 1:1 mixture of PBS:matrigel to a final concentration of 1 × 10^8 ^cells ml^−1^. In total, 10 million wild-type DFFB and DMEM-KO A375 cells were subcutaneously injected into opposing flanks of 6–8-week-old female NSG mice (Jackson Laboratory, 005557). Mice were housed up to five animals per ventilator cage with ad libitum food and water on a 12-h light–dark cycle maintained in an ambient temperature of 67–74 °F and 40–70% humidity. When tumour volumes reached 100–200 mm^3^, drug dosing was started via once daily oral gavage with 100 mg kg^−1^ dabrafenib and 1 mg kg^−1^ trametinib. Tumour volumes were measured one to two times per week by blinded observers. Tumour volumes were normalized to the minimum volume of each tumour during treatment. Only mice with at least one relapsed tumour (wild-type or knockout), defined by tenfold regrowth from the minimal volume during treatment, were included in Fig. 3f. Additional mice which did not exhibit tumour relapse of either wild-type or knockout tumours were excluded from Fig. 3f but are included in the Fig. 3 source data. Mice were killed upon reaching the maximal allowed tumour burden of 2,000 mm^3^.

### RNA extraction and quantitative RT–PCR

RNA from A375 parental cells and cells treated with 1 μM dabrafenib and 100 nM trametinib with and without 10 μM QVD for 6 days was extracted with TRIzol (Thermo Fisher, 15596026) following the manufacturer’s protocol. RNA was converted to cDNA with the RevertAid First Strand cDNA Synthesis Kit (Thermo Fisher, K1621) before quantifying *IFNB1* levels with TaqMan assay (Thermo Fisher, 4331182).

### Bulk RNA-seq analysis

Biological triplicates of A375 wild-type, DFFB-KO cl1 and ATF3-depleted (pooled KO) parental cells, persister cells and persister cells cotreated for the duration of persister formation with 20 μM AP1 inhibitor T-5224 were trypsinized and RNA was isolated with the RNeasy Mini Kit (Qiagen, 74104). The libraries were constructed with Illumina Stranded mRNA Prep and sequenced using a NovaSeq S4 flowcell on an Illumina NovaSeq 6000 sequencer at the UCSD Institute for Genomic Medicine. Analysis was performed using Partek Flow software (v12.1.0) with default options, unless otherwise noted. Reads were aligned to human hg38 reference genome with STAR aligner, v2.7.8a. The Partek task ‘quantify to annotation model’ quantified reads with the Ensembl 112 release. Differentially expressed genes were calculated with DESeq2 using default settings (v1.46.0), and gene set enrichment analysis was performed with clusterProfiler (v4.14.6). The Benjamini–Hochberg method was used for false discovery rate correction.

### Cytosolic mitochondrial DNA isolation and PCR

Cytosolic DNA was isolated as previously described^70^. In brief, cells from each condition were split into two pellets. For the whole-cell fraction, DNA was extracted from the first pellet with the DNeasy Blood and Tissue Kit (Qiagen, 69504). For the cytosolic fraction, the second pellet was resuspended in 150 mM NaCl, 50 mM HEPES pH 7.4 and 25 μg ml^−1^ digitonin for 10 min at room temperature. This mixture was centrifuged (150*g*) at 4 °C for 10 min and supernatant serially collected three times. The supernatant was then centrifuged (17,000*g*) at 4 °C for 10 min, and the pellet DNA was cleaned with the Qiaquick Nucleotide Removal Kit (Qiagen, 28115). All DNA was quantified by spectrophotometry. The primers in Supplementary Table 19 were used for PCR with PowerTrack SYBR Green Master Mix (Thermo Fisher, A46012). The cytosolic fraction measurements were normalized to the respective whole-cell fraction measurement.

### Statistics and reproducibility

All statistical tests were performed with GraphPad Prism version 9.0.0 (86), R version 4.0.3 or RStudio version 1.2.5033. No statistical methods were used to predetermine sample sizes, but our sample sizes are similar to those reported in previous publications^7,25^. No data were excluded from the analyses unless otherwise stated. The experiments were not randomized. The investigators were not blinded to allocation during experiments and outcome assessment unless otherwise stated. The data distribution was assumed to be normal, but this was not formally tested. For the *P* values, non significant (n.s.) indicates *P* > 0.05. Differentially expressed genes from single-cell RNA-seq were calculated using the Wilcoxon rank-sum test. Differentially expressed genes from bulk RNA-seq data were calculated using the Wald test in DESeq2. Gene set enrichment analysis was performed using a Kolmogorov–Smirnov-like statistic. The signature scoring significance between selected populations was calculated with the two-sided Mann–Whitney test. Multiple testing correction was performed using the Benjamini–Hochberg method. All experiments were repeated at least two times except for the data in Fig. 3i, Extended Data Fig. 2j and the RNA-seq and whole-exome sequencing experiments.

### Reporting summary

Further information on research design is available in the Nature Portfolio Reporting Summary linked to this article.

## Online content

Any methods, additional references, Nature Portfolio reporting summaries, source data, extended data, supplementary information, acknowledgements, peer review information; details of author contributions and competing interests; and statements of data and code availability are available at 10.1038/s41556-025-01810-x.

## Supplementary information

Supplementary Information Supplementary Figs. 1–6.

Reporting summary

Supplementary Table 1 Differentially expressed genes from bulk RNA-seq of A375 WT persister versus parental cells.

Supplementary Table 2 Enriched Hallmarks gene sets from bulk RNA-seq of A375 WT persister versus parental cells.

Supplementary Table 3 Genes in the anastasis gene set.

Supplementary Table 4 Whole exome sequencing acquired mutation list from drug-treated A375 WT and DFFB KO cells.

Supplementary Table 5 Whole exome sequencing acquired mutation list from drug-treated PC9 WT and DFFB KO cells.

Supplementary Table 6 Differentially expressed genes from single-cell RNA-seq of A375 DFFB WT versus KO cells at the persister and DTEP timepoints.

Supplementary Table 7 Enriched Hallmarks gene sets from single-cell RNA-seq of A375 DFFB WT versus KO cells at the persister and DTEP timepoints.

Supplementary Table 8 Differentially expressed genes from bulk RNA-seq of A375 DFFB KO versus WT persister cells.

Supplementary Table 9 Enriched Hallmarks gene sets from bulk RNA-seq of A375 DFFB KO versus WT persister cells.

Supplementary Table 10 Differentially expressed genes from bulk RNA-seq of A375 ATF3-depleted versus WT persister cells.

Supplementary Table 11 Enriched Hallmarks gene sets from bulk RNA-seq of A375 ATF3-depleted versus WT persister cells.

Supplementary Table 12 Differentially expressed genes from bulk RNA-seq of A375 ATF3-depleted persister cells treated with versus without AP1 inhibitor.

Supplementary Table 13 Enriched Hallmarks gene sets from bulk RNA-seq of A375 ATF3-depleted persister cells treated with versus without AP1 inhibitor.

Supplementary Table 14 Differentially expressed genes from bulk RNA-seq of A375 DFFB KO persister cells treated with versus without AP1 inhibitor.

Supplementary Table 15 Enriched Hallmarks gene sets from bulk RNA-seq of A375 DFFB KO persister cells treated with versus without AP1 inhibitor.

Supplementary Table 16 Differentially expressed genes from bulk RNA-seq of A375 WT persister cells treated with versus without AP1 inhibitor.

Supplementary Table 17 Enriched Hallmarks gene sets from bulk RNA-seq of A375 WT persister cells treated with versus without AP1 inhibitor.

Supplementary Table 18 Antibody sources.

Supplementary Table 19 Primer sequences.

## Source data

Source Data Fig. 1 Statistical source data.

Source Data Fig. 3 Statistical source data.

Source Data Fig. 4 Statistical source data.

Source Data Fig. 5 Statistical source data.

Uncropped westerns Unprocessed western blots.

Source Data Extended Data Fig. 2 Statistical source data.

Source Data Extended Data Fig. 3 Statistical source data.

Source Data Extended Data Fig. 4 Statistical source data.

Source Data Extended Data Fig. 5 Statistical source data.

Source Data Extended Data Fig. 6 Statistical source data.

Source Data Extended Data Fig. 7 Statistical source data.

Source Data Extended Data Fig. 8 Statistical source data.

Source Data Extended Data Fig. 9 Statistical source data.

Source Data Extended Data Fig. 10 Statistical source data.

## Data Availability

All data are available from the authors without restriction. Single-cell and bulk RNA-seq data have been deposited in NCBI’s Gene Expression Omnibus (GEO) (accession number: GSE196018). Whole-exome and whole-genome sequencing data have been deposited in the NCBI Sequence Read Archive (PRJNA800470). Previously published data that were reanalysed here are available under accession codes PRJNA591860 for lung cancer patient single-cell RNA-seq data and EGA S00001000992 in the European Genome-phenome Archive for melanoma patient RNA-seq data. Source data are provided with this paper.
