# Supplementary material for: DNA fragmentation factor B suppresses interferon to enable cancer persister cell regrowth
Source: Nat Cell Biol. 2025 Nov 17;27(12):2143–51. doi: 10.1038/s41556-025-01810-x (PMC12717002; doi:10.1038/s41556-025-01810-x)
Supplement: Supplementary file 1 — Supplementary Figs. 1–6. [file 41556_2025_1810_MOESM1_ESM.pdf]

nature cell biology

Supplementary information https://doi.org/10.1038/s41556-025-01810-x

# DNA fragmentation factor B suppresses interferon to enable cancer persister cell regrowth

In the format provided by the authors and unedited

**Supplementary Information**

Table of Contents:

Supplementary Figure 1: Flow cytometry schematic for mitochondrial release of cytochrome c assay.

Supplementary Figure 2: Flow cytometry schematic for JC-1 indicator dye-based assessment of loss of mitochondrial potential.

Supplementary Figure 3: Cleaved caspase 3 flow cytometry schematic.

Supplementary Figure 4: Caspase 3/7 activity reporter flow cytometry schematic.

Supplementary Figure 5: γH2AX DNA damage flow cytometry schematic.

Supplementary Figure 6: Flow cytometry schematic for mitochondrial release of cytochrome c with BH3 mimetics treatment assay.

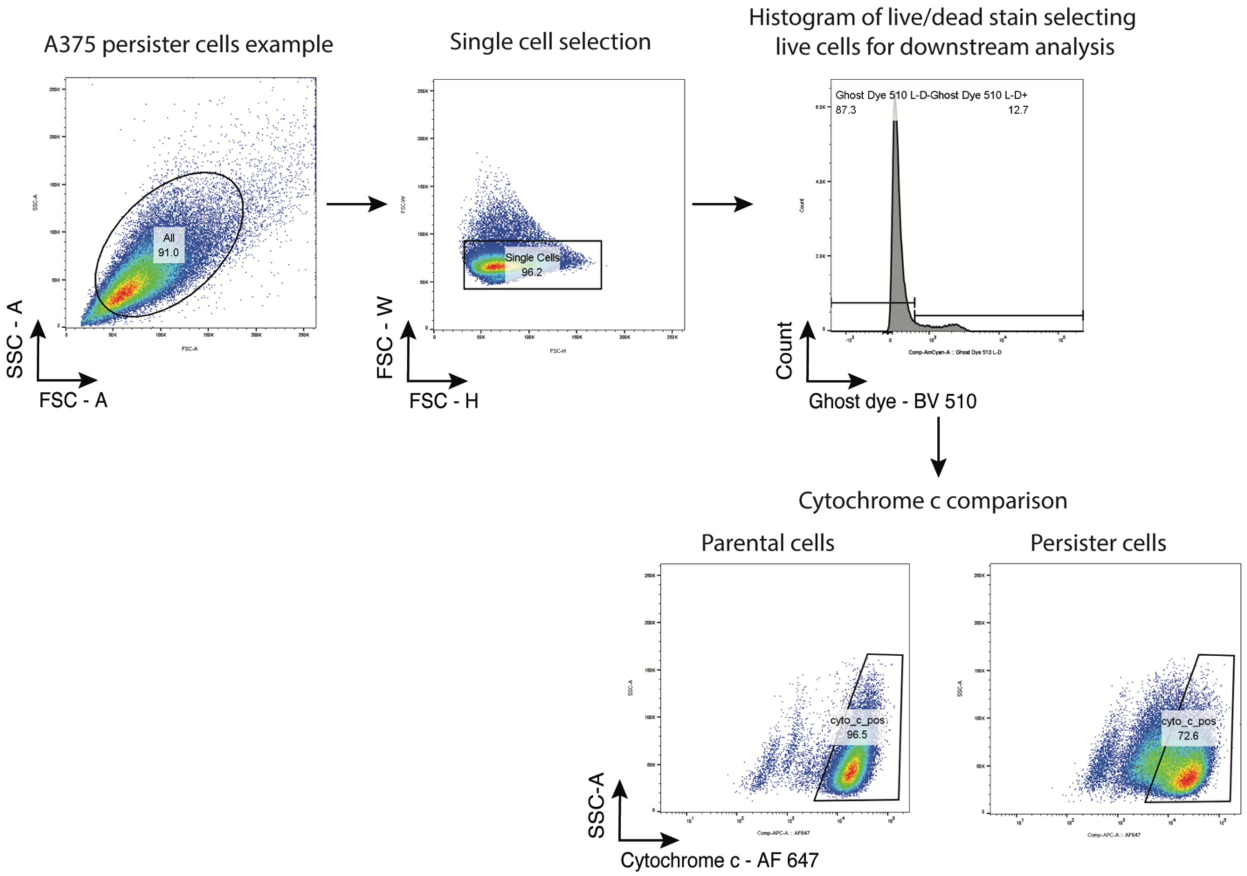


**Supplementary Figure 1: Flow cytometry schematic for mitochondrial release of cytochrome c assay.** Gating strategy used for A375 parental cells and persister cells derived from treatment with 250 nM dabrafenib and 25 nM trametinib in Figure 1g. See methods for details of this assay.

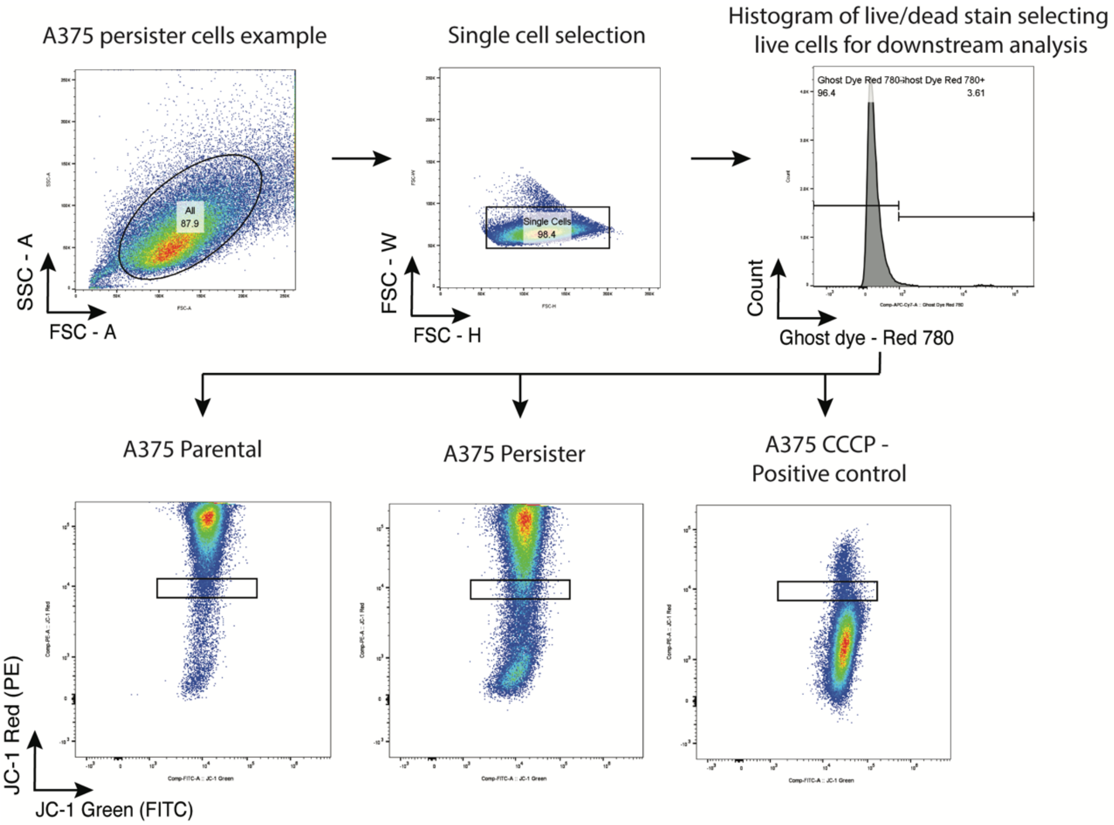


**Supplementary Figure 2: Flow cytometry schematic for JC-1 indicator dye-based assessment of loss of mitochondrial potential.** Gating strategy used for Extended Data Figure 3a. Representative flow cytometry gating for loss of mitochondrial potential in A375 parental cells and persister cells treated with 250 nM dabrafenib and 25 nM trametinib. Results from triplicate biological replicates are presented in Fig. 1h. For the positive control, cells were treated with mitochondrial oxidative phosphorylation uncoupler carbonyl cyanide m-chlorophenylhydrazone (CCCP) (50 µM) for 5 minutes. Gating was set to compare relative levels of incomplete loss of mitochondrial potential, indicated by intermediate levels of loss of JC-1 red fluorescence, as illustrated.

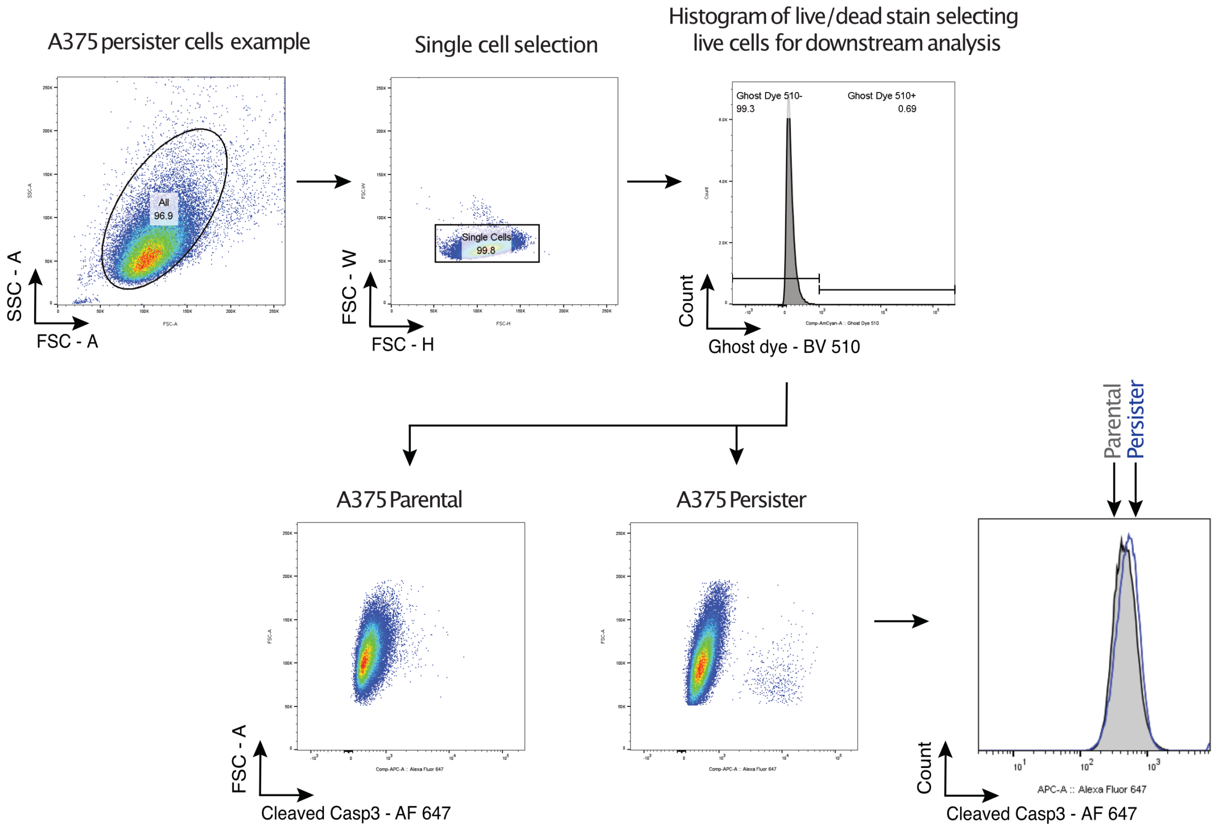


**Supplementary Figure 3: Cleaved caspase 3 flow cytometry schematic.** Gating strategy used for A375 parental cells and persister cells derived from treatment with 250 nM dabrafenib and 25 nM trametinib in Extended Data Figure 2a.

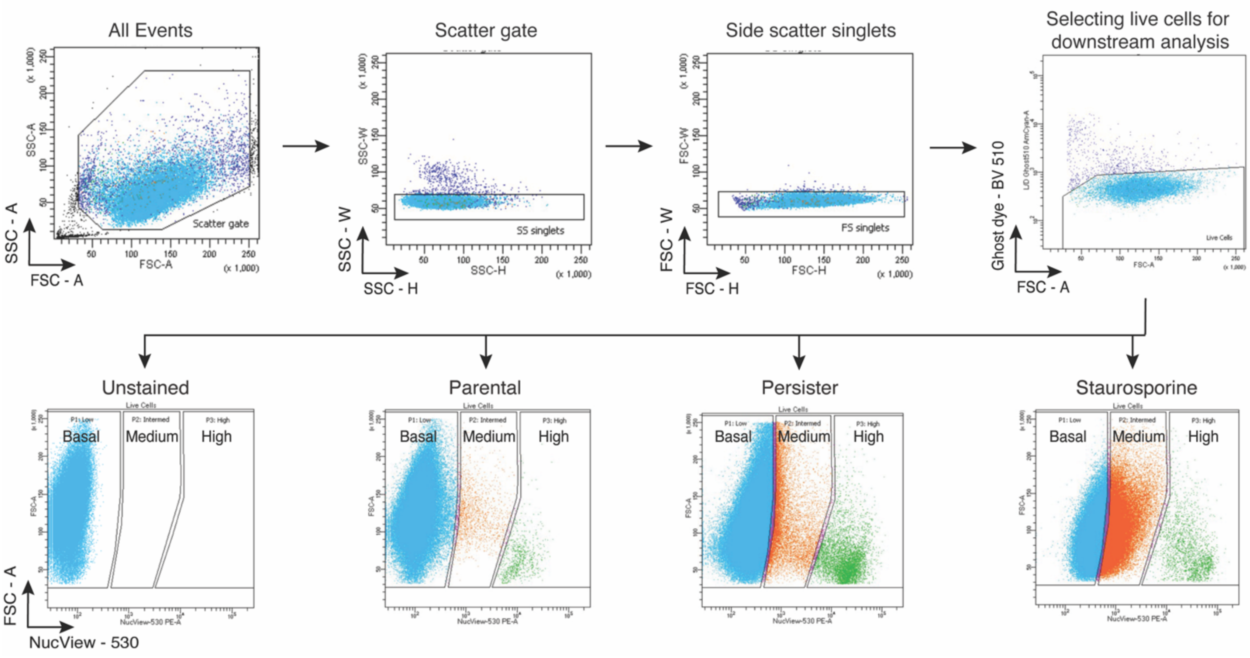


**Supplementary Figure 4: Caspase 3/7 activity reporter flow cytometry schematic. a**, Representative gating strategy for sorting caspase 3/7 activity sensor levels in A375 parental cells, persister cells treated with 250 nM dabrafenib and 25 nM trametinib, and staurosporine-treated positive control cells. Representative caspase 3/7 activity sensor levels for each condition shown in bottom row. This gating strategy was used to sort the Basal, Medium and High Caspase 3/7 activity level persister cell populations for Figure 1j,k and Extended Data Figure 2b,j.

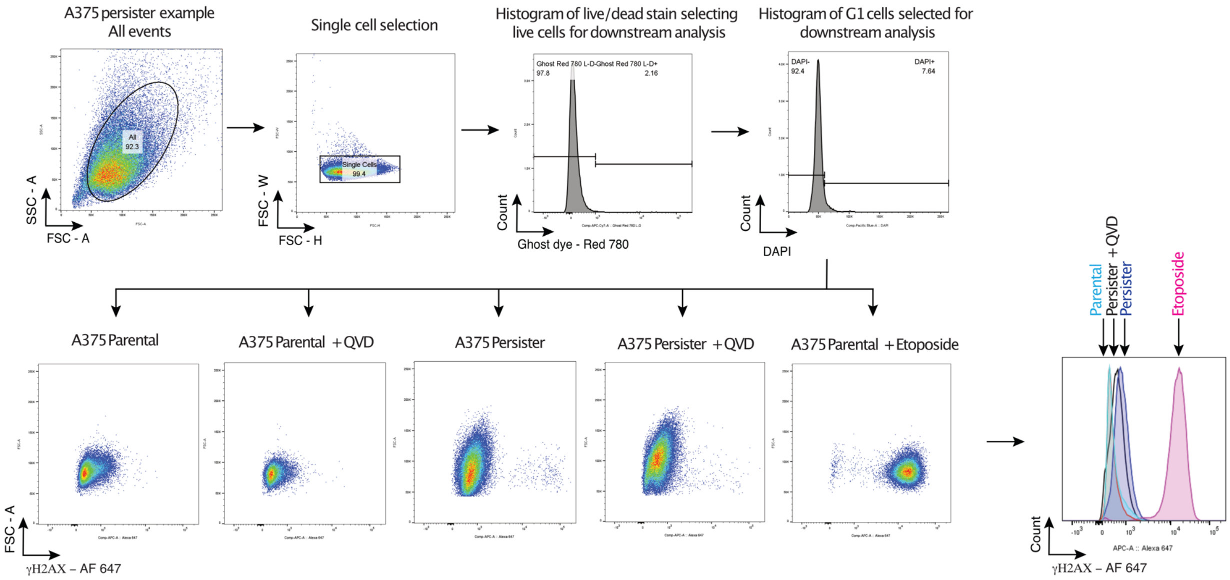


**Supplementary Figure 5: γH2AX DNA damage flow cytometry schematic.** Representative flow cytometry gating for γH2AX in A375 parental cells, persister cells treated with 250 nM dabrafenib and 25 nM trametinib, and positive control etoposide-treated cells. 10 µM QVD co-treatment was present for the duration of the experiment for QVD treated cells. Parental cell treatment with 100 µM etoposide for 30 minutes was used as a positive control. This gating was used for Extended Data Fig. 2h.

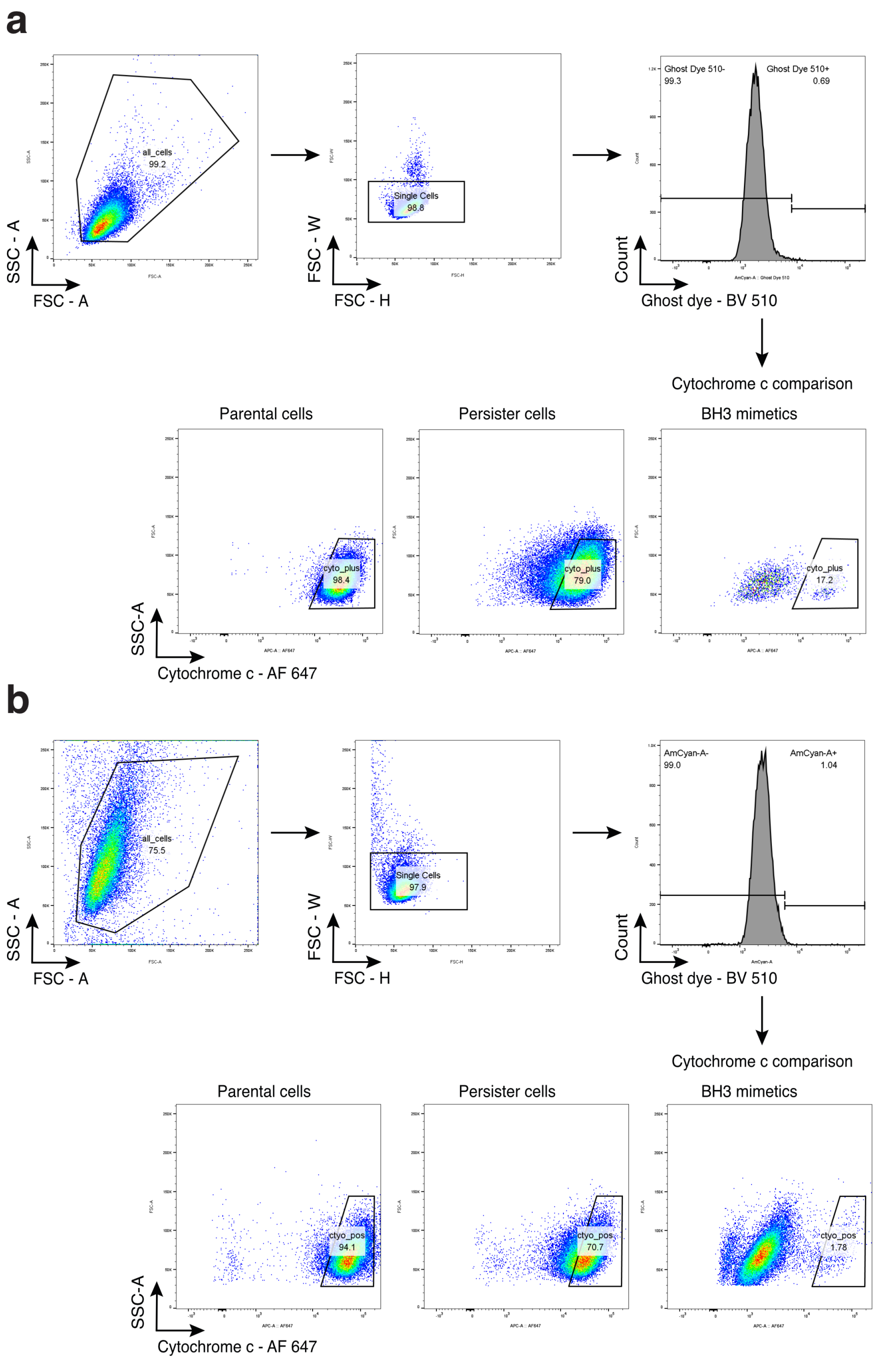


**Supplementary Figure 6: Flow cytometry schematic for mitochondrial release of cytochrome c with BH3 mimetics treatment assay.** Gating strategy used for A375 (**a**) and PC9 (**b**) parental, BH3 mimetic-treated, and persister cells in Extended Data Fig. 8c-f. See methods for details of this assay.
