# Supplementary material for: DNA fragmentation factor B suppresses interferon to enable cancer persister cell regrowth
Source: Nat Cell Biol. 2025 Nov 17;27(12):2143–51. doi: 10.1038/s41556-025-01810-x (PMC12717002; doi:10.1038/s41556-025-01810-x)
Supplement: Supplementary file 2 — Reporting summary [file 41556_2025_1810_MOESM2_ESM.pdf]

nature portfolio

Corresponding author(s): Matthew Hangauer

Last updated by author(s): Oct 9, 2025

Reporting Summary

Nature Portfolio wishes to improve the reproducibility of the work that we publish. This form provides structure for consistency and transparency in reporting. For further information on Nature Portfolio policies, see our Editorial Policies and the Editorial Policy Checklist.

Statistics

For all statistical analyses, confirm that the following items are present in the figure legend, table legend, main text, or Methods section.

n/a	Confirmed	
☐	☒	The exact sample size (*n*) for each experimental group/condition, given as a discrete number and unit of measurement
☐	☒	A statement on whether measurements were taken from distinct samples or whether the same sample was measured repeatedly
☐	☒	The statistical test(s) used AND whether they are one- or two-sided<br>*Only common tests should be described solely by name; describe more complex techniques in the Methods section.*
☐	☒	A description of all covariates tested
☐	☒	A description of any assumptions or corrections, such as tests of normality and adjustment for multiple comparisons
☐	☒	A full description of the statistical parameters including central tendency (e.g. means) or other basic estimates (e.g. regression coefficient) AND variation (e.g. standard deviation) or associated estimates of uncertainty (e.g. confidence intervals)
☐	☒	For null hypothesis testing, the test statistic (e.g. *F*, *t*, *r*) with confidence intervals, effect sizes, degrees of freedom and *P* value noted<br>*Give P values as exact values whenever suitable.*
☒	☐	For Bayesian analysis, information on the choice of priors and Markov chain Monte Carlo settings
☒	☐	For hierarchical and complex designs, identification of the appropriate level for tests and full reporting of outcomes
☒	☐	Estimates of effect sizes (e.g. Cohen's *d*, Pearson's *r*), indicating how they were calculated

Our web collection on statistics for biologists contains articles on many of the points above.

Software and code

Policy information about availability of computer code

Data collection	NovaSeq 6000 Sequencer (RNA sequencing, whole exome and whole genome sequencing). Molecular Devices SpectraMax iD3 plate reader (Cell viability). EVOS XL Core microscope (microscopy). Agilent TapeStation (cDNA library quality control). BD FACSCanto RUO flow cytometer. (Flow cytometry - JC-1 mitochondrial depolarization, cytochrome c release). BD FACSAria II flow cytometry sorter (Caspase 3/7 activity). iBLOT 2 Dry Blotting System (Western blot transfer). LICOR Odyssey Imaging System (Western blot imaging).
Data analysis	10X Genomics Cell Ranger version 3.1.0 (scRNAseq). Seurat R package version 4.0.3 (scRNAseq). ClusterProfiler R package version 4.14.6 (sc and bulk RNAseq). AUCell package version 1.12.0 (scRNAseq). Partek Flow Software version 12.1.0. (bulk RNAseq). DESeq2 version 1.46.0. FlowJo™ Software (BD Life Sciences) version 10.7.1 (flow cytometry). Image Studio version 5.2 (Western blot). SoftMax Pro version 7.0.3 (Plate reader - cell viability). GATK version 4.2.4.0 pipelines (whole exome and whole genome sequencing). Maftools R Bioconductor package version 2.6.05 (whole exome sequencing). PICKLES version 3 (DFFB essentiality across cell lines). GraphPad Prism version 9.0.0 (graphs and statistical analysis). R version 4.0.3 (graphs, statistical analysis, RNAseq analysis). RStudio version 1.2.5033 (graphs, statistical analysis, RNAseq analysis, WES analysis). Affinity Designer version 2.5.3 (figures).

For manuscripts utilizing custom algorithms or software that are central to the research but not yet described in published literature, software must be made available to editors and reviewers. We strongly encourage code deposition in a community repository (e.g. GitHub). See the Nature Portfolio guidelines for submitting code & software for further information.

Data

Policy information about availability of data

All manuscripts must include a data availability statement. This statement should provide the following information, where applicable:

- Accession codes, unique identifiers, or web links for publicly available datasets
- A description of any restrictions on data availability
- For clinical datasets or third party data, please ensure that the statement adheres to our policy

Single cell and bulk RNA sequencing data have been deposited in NCBI's Gene Expression Omnibus (accession number: GSE196018). Whole exome sequencing data have been deposited in the NCBI Sequence Read Archive (PRJNA800470). DFFB essentiality scores from the PICKLES database is available from the Hart laboratory (https://pickles.hart-lab.org/). Human lung cancer data were extracted from data in Maynard et al. Cell 2020, PMID: 32822576. Human melanoma data were extracted from data in Kwong et al. J Clin. Invest. 2015, PMID: 25705882.

Field-specific reporting

Please select the one below that is the best fit for your research. If you are not sure, read the appropriate sections before making your selection.

☒ Life sciences ☐ Behavioural & social sciences ☐ Ecological, evolutionary & environmental sciences

For a reference copy of the document with all sections, see nature.com/documents/nr-reporting-summary-flat.pdf

Life sciences study design

All studies must disclose on these points even when the disclosure is negative.

Sample size	We used at least 3 biological replicates for all experiments except the scRNAseq experiment.
Data exclusions	For scRNAseq analysis: cells were excluded for quality control as described in the methods section. For WES analysis: low variant allele frequency mutations were excluded as defined in the methods to focus on mutations that were acquired during drug treatment.
Replication	All experiments were repeated at least two times except for data in Fig. 4i, Extended Data Fig. 2j, and the RNAseq and WES experiments.
Randomization	Randomization of mice is not relevant for the reported mouse experiments because both experimental conditions (WT and KO tumours) were included on opposing flanks of each mouse.
Blinding	All DTEP colony formation experiments were blinded prior to counting and in vivo tumor measurements were taken by blinded observers.

Reporting for specific materials, systems and methods

We require information from authors about some types of materials, experimental systems and methods used in many studies. Here, indicate whether each material, system or method listed is relevant to your study. If you are not sure if a list item applies to your research, read the appropriate section before selecting a response.

Materials & experimental systems

n/a	Involved in the study	
☐	☒	Antibodies
☐	☒	Eukaryotic cell lines
☒	☐	Palaeontology and archaeology
☐	☒	Animals and other organisms
☒	☐	Human research participants
☒	☐	Clinical data
☒	☐	Dual use research of concern

Methods

n/a	Involved in the study	
☒	☐	ChIP-seq
☐	☒	Flow cytometry
☒	☐	MRI-based neuroimaging

Antibodies

Antibodies used	B-Tubulin (Invitrogen, MA5-16308); Vinculin (Cell Signaling Technology, #4650); γH2AX (Cell Signaling Technology, #9718); Cleaved caspase 3 (Cell Signaling Technology, #9664); STAT1 (Cell Signaling Technology, #9176); phosphorylated STAT1 (Cell Signaling Technology, #9167); phosphorylated TBK1 (Cell Signaling Technology, #5483); phosphorylated IRF3 (Cell Signaling Technology, #37829); ATF3 (Cell Signaling Technology, #33593); STING (Cell Signaling Technology, #13647); Caspase 9 (Cell Signaling Technology, #9502); Cytochrome c (BioLegend, #612310); DFFB (Santa Cruz Biotechnology, SC-374067); DFFA (Abcam, ab108521); ATF4 (Cell Signaling Technology, #11815); phosphorylated eIF2a (Cell Signaling Technology, #3398); eIF2a (Cell Signaling Technology, #5324); IRDye 680RD Goat anti-Mouse IgG secondary antibody (LICOR, #926-68070); IRDye 800CW Goat anti-Mouse IgG secondary antibody

(LICOR, #926-32210); IRDye 680RD Goat anti-Rabbit IgG secondary antibody (LICOR, #926-68071); IRDye 800CW Goat anti-Rabbit IgG secondary antibody (LICOR, #926-32211).

Validation	All antibodies are commercially available and used at dilutions recommended by manufacturer. B-Tubulin (Invitrogen, MA5-16308; https://www.thermofisher.com/antibody/product/beta-Tubulin-Loading-Control-Antibody-clone-BT7R-Monoclonal/MA5-16308). yH2AX (Cell Signaling Technology, #9718; https://www.cellsignal.com/products/primary-antibodies/phospho-histone-h2a-x-ser139-20e3-rabbit-mab/9718). Cleaved Caspase-3 (Cell Signaling Technology, #9664; https://www.cellsignal.com/products/primary-antibodies/cleaved-caspase-3-asp175-5a1e-rabbit-mab/9664). DFFB (Santa Cruz Biotechnology, SC-374067; https://www.scbt.com/p/cad-antibody-f-11). STING (Cell Signaling Technology, #13647; https://www.cellsignal.com/products/primary-antibodies/sting-d2p2f-rabbit-mab/13647). Cytochrome c (BioLegend, #612310; https://www.biolegend.com/nl-be/products/alexa-fluor-647-anti-cytochrome-c-antibody-10789). DFFA (Abcam, ab108521; https://www.abcam.com/icad-antibody-epr42222-ab108521.html). IRDye 680RD Goat anti-Mouse IgG secondary antibody (LICOR, #926-68070; https://www.licor.com/bio/reagents/irdye-680rd-goat-anti-mouse-igg-secondary-antibody). IRDye 800CW Goat anti-Mouse IgG secondary antibody (LICOR, #926-32210; https://www.licor.com/bio/reagents/irdye-800cw-goat-anti-mouse-igg-secondary-antibody). IRDye 680RD Goat anti-Rabbit IgG secondary antibody (LICOR, #926-68071; https://www.licor.com/bio/reagents/irdye-680rd-goat-anti-rabbit-igg-secondary-antibody). IRDye 800CW Goat anti-Rabbit IgG secondary antibody (LICOR, #926-32211; https://www.licor.com/bio/reagents/irdye-800cw-goat-anti-rabbit-igg-secondary-antibody). Vinculin (Cell Signaling Technology, #4650; https://www.cellsignal.com/products/primary-antibodies/vinculin-antibody/4650). Ghost Dye Red 780 (Tonbo Biosciences, #13-0865-T100; https://tonbobio.com/products/ghost-dye-red-780). STAT1 (Cell Signaling Technology, #9176; https://www.cellsignal.com/products/primary-antibodies/stat1-9h2-mouse-mab/9176); phosphorylated STAT1 (Cell Signaling Technology, #9167; https://www.cellsignal.com/products/primary-antibodies/phospho-stat1-tyr701-58d6-rabbit-mab/9167); phosphorylated TBK1 (Cell Signaling Technology, #5483; https://www.cellsignal.com/products/primary-antibodies/phospho-tbk1-nak-ser172-d52c2-xp-rabbit-mab/5483); phosphorylated IRF3 (Cell Signaling Technology, #37829; https://www.cellsignal.com/products/primary-antibodies/phospho-irf-3-ser386-e7j8g-xp-174-rabbit-mab/37829); ATF3 (Cell Signaling Technology, #33593; https://www.cellsignal.com/products/primary-antibodies/atf-3-d2y5w-rabbit-mab/33593); Caspase 9 (Cell Signaling Technology, #9502; https://www.cellsignal.com/products/primary-antibodies/caspase-9-antibody/9502); ATF4 (Cell Signaling Technology, #11815; https://www.cellsignal.com/products/primary-antibodies/atf-4-d4b8-rabbit-mab/11815?srsltid=AfmBOoqE-nDZWY-2noWj6UWXU9EG7CrJxJxg1kRSYQI-EoiDCabd5GQc); phosphorylated eIF2a (Cell Signaling Technology, #3398; https://www.cellsignal.com/products/primary-antibodies/phospho-eif2a-ser51-d9g8-xp-rabbit-mab/3398?srsltid=AfmBOooGIlNOKybTM26vwTXqU6KpwmENDwyN3njH7GfPKoJKI2p9Xbfs); eIF2a (Cell Signaling Technology, #5324; https://www.cellsignal.com/products/primary-antibodies/eif2a-d7d3-xp-rabbit-mab/5324?srsltid=AfmBOop9cnstqtunbhBpquA71M1IDE6H4iqBXlJTB7QGuLL75FGpuEqv).

Eukaryotic cell lines

Policy information about cell lines

Cell line source(s)	A375 (CRL-1619) and BT474 (HTB-20) cells were purchased from the ATCC. PC9 cells were provided by the Altschuler and Wu Lab at UC San Francisco as stated in the methods section.
Authentication	Cell line identities were confirmed with STR profiling at the UC Berkeley Cell Culture Facility.
Mycoplasma contamination	All cell lines regularly tested negative for mycoplasma throughout these investigations using the Lonza Mycoalert Mycoplasma Detection Kit.
Commonly misidentified lines (See ICLAC register)	None of the cell lines used are commonly misidentified.

Animals and other organisms

Policy information about studies involving animals; ARRIVE guidelines recommended for reporting animal research

Laboratory animals	6-8 week old female NSG mice (Jackson Laboratory, #005557)
Wild animals	The study did not involve wild animals.
Field-collected samples	The study did not involve samples collected from the field.
Ethics oversight	The University of California San Francisco Institutional Animal Care and Use Committee (IACUC) approved the xenograft studies which were performed at the UCSF Preclinical Therapeutics Core in protocol #AN179937.

Note that full information on the approval of the study protocol must also be provided in the manuscript.

Flow Cytometry

Plots

Confirm that:

☒ The axis labels state the marker and fluorochrome used (e.g. CD4-FITC).

☒ The axis scales are clearly visible. Include numbers along axes only for bottom left plot of group (a 'group' is an analysis of identical markers).

☒ All plots are contour plots with outliers or pseudocolor plots.

☒ A numerical value for number of cells or percentage (with statistics) is provided.

Methodology

Sample preparation	JC-1 mitochondrial membrane potential assay<br>A375 persister cells were derived with 250 nM dabrafenib and 25 nM trametinib for 2 weeks and untreated parental cells were cultured alongside. Cells were then lifted with trypsin for JC-1 staining. As a positive control, cells were treated with 50 µM carbonyl cyanide m-chlorophenyl hydrazone (CCCP) for 5 minutes. JC-1 was dissolved in DMSO (Thermo Fisher Scientific, #D12345) at a final concentration of 1.5 µM and cells were stained for 30 minutes away from light at 37 °C. Cells were then stained with Ghost Dye Red 780 (Tonbo Biosciences, #13-0865-T100) diluted 1:1000 in PBS (Gibco, #10010023) for 15 minutes away from light at room temperature. Cells were washed in PBS and analyzed on a BD FACSCanto RUO flow cytometer using 488 nm and 640 nm lasers for green and red fluorescence respectively. At least 30,000 live cell events were collected per sample. The flow cytometry results were analyzed using FlowJo Software (BD Life Sciences) version 10.7.1. See Supplementary Fig. 3 for gating strategy.<br><br>DNA damage, cleaved caspase 3, and cytochrome c flow cytometry<br>A375 persister cells were derived with 250 nM dabrafenib and 25 nM trametinib for 2 weeks and untreated parental cells were cultured alongside. For γH2AX experiments, A375 persister cells treated with 10 µM QVD for the duration of drug treatment were also prepared. After 2 weeks of culture, cells were trypsinized and collected for staining. Cells were stained with viability dye Ghost Dye Red 510 or 780 diluted 1:1000 in PBS for 15 minutes away from light at room temperature. For cytochrome c staining, incubation in 50 µg/mL digitonin in PBS for 10 minutes on ice was performed to selectively permeabilize the cell membrane.63 Cells were fixed with 4% paraformaldehyde for 10 minutes at room temperature. For γH2AX and cleaved caspase 3 staining, cells were permeabilized with 0.3% Triton X-100 in PBS for 10 minutes at room temperature. Cells were stained for proteins of interest using primary conjugated antibodies at manufacturer recommended dilutions in PBS for 30 minutes to 1 hour at room temperature. Cells were washed in PBS and analyzed on a BD FACSCanto RUO flow cytometer using 405 nm and 640 nm lasers for blue and red fluorescence respectively. At least 30,000 live cell events were collected per sample. Flow cytometry data were analyzed using FlowJo Software (BD Life Sciences) version 10.7.1. See Supplementary Figs. 2 and 7 for cytochrome c flow cytometry, Supplementary Fig. 4 for cleaved caspase 3 gating strategy, and Supplementary Fig. 6 for γH2AX gating strategy.<br><br>Caspase 3/7 activity reporter assay<br>Caspase activity was measured using NucView 530 Caspase-3 Substrate (Biotium #10408). A375 persister cells were pre-derived from 2 week treatment with 250 nM dabrafenib with 25 nM trametinib while parental cells were cultured alongside. Positive control cells were derived from 4-hour parental cell treatment with 1 µM staurosporine and these pre-apoptotic cells were assayed by flow cytometry prior to death. Adherent tumour cells were collected by trypsinization (~1 million cells/ sample), washed in PBS, and incubated with Ghost Dye Violet 510 cell viability dye following the manufacturer's instructions. Cells were washed with PBS + 1% FBS and subsequently incubated with 5 µM NucView 530 Caspase-3 Substrate for 30 minutes in PBS + 1% FBS and analyzed by flow cytometry using a BD FACSAria II sorter. Cells not treated with NucView 530 Caspase-3 Substrate were used as unstained controls. Fluorescence was measured as follows: Ghost Dye Violet 510 (405 nm laser, 525/50 filter), NucView 530 Caspase-3 Substrate (488 laser, 530/30 filter). See Supplementary Fig. 5 for gating strategy. Sorted live cells were collected in PBS + 10% FBS and used for downstream viability assays. For testing caspase 3/7 activity-positive persister cell regrowth, cells were sorted and plated at 1,000 cells per well in 12-well plates in drug-free media. 24 hours later, CTG was performed to measure cell viability. To assess regrowth ability, drug-free media was refreshed on day 3 and CTG was performed on day 6. The DNA damage levels for A375 persister cells with basal and medium caspase activity were measured by sorting cells and extracting protein for western blot analysis as described in the methods section.
Instrument	BD FACSCanto RUO (cytochrome c, JC-1, cleaved caspase 3, gH2AX) and BD FACSAria II (caspase 3/7 activity).
Software	The flow cytometry results were analyzed using FlowJo™ Software (BD Life Sciences) version 10.7.1.
Cell population abundance	At least 30,000 live cell events were collected per sample. Live cells were determined using the Ghost Dye Red 780 (Tonbo Biosciences, #13-0865-T100) or Ghost Dye Violet 510 (Tonbo, #13-0870-T100).
Gating strategy	Live (x = SSC-A, y = FSC-A) and singlet (x = FSC-W, y = FSC-H) gates were used. Live cells were further confirmed with Ghost Dye Red 780/ Violet 510 (x = FSC-A, y = Ghost dye - Red 780/Violet 510). Each respective condition was then analyzed. See Supplementary Figures 1-6 for all gating strategy schematics.

☒ Tick this box to confirm that a figure exemplifying the gating strategy is provided in the Supplementary Information.
