# Supplementary material for: DNA fragmentation factor B suppresses interferon to enable cancer persister cell regrowth
Source: Nat Cell Biol. 2025 Nov 17;27(12):2143–51. doi: 10.1038/s41556-025-01810-x (PMC12717002; doi:10.1038/s41556-025-01810-x)

Unprocessed western blot images.

Fig 1i

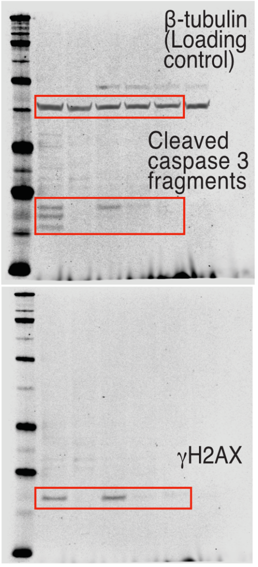

Fig 1l

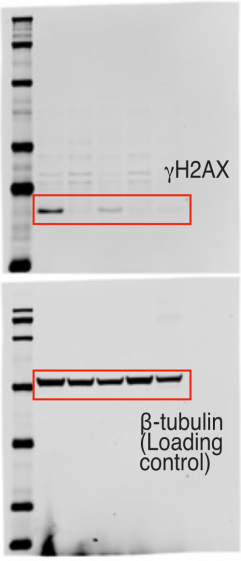

Fig 2a

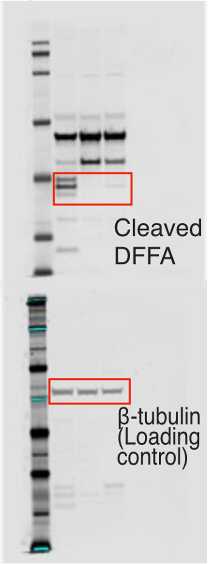

Fig 2b

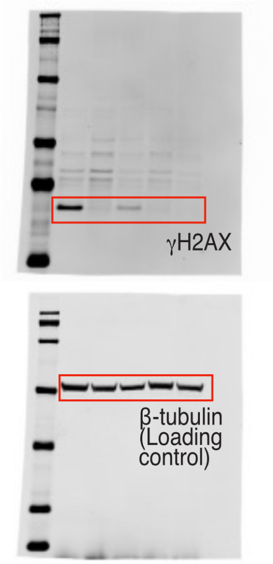

Fig 2c

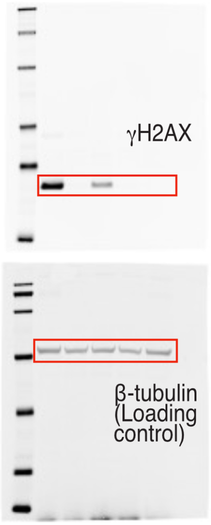

Fig 2d

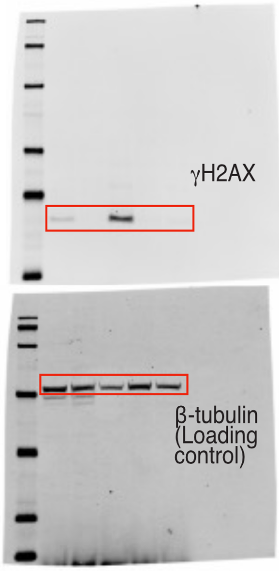

Fig 2e

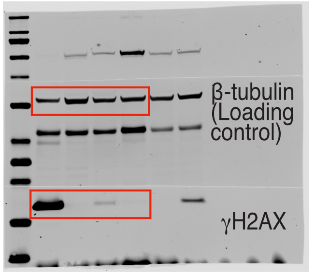

Fig 2f

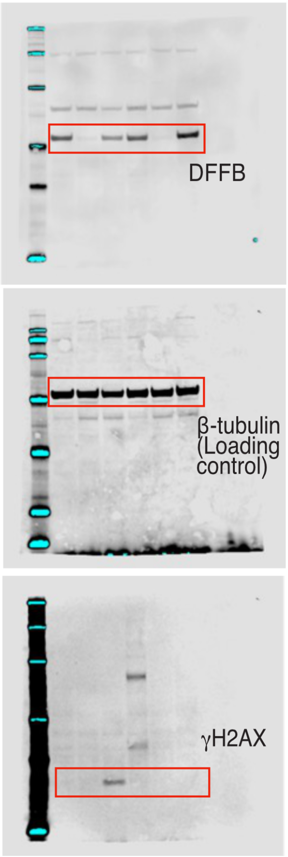

Fig 4f

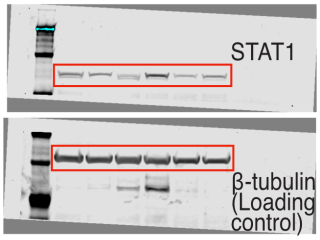

Fig 4g

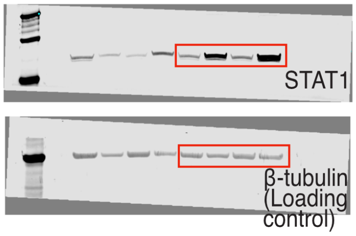

Fig 4h

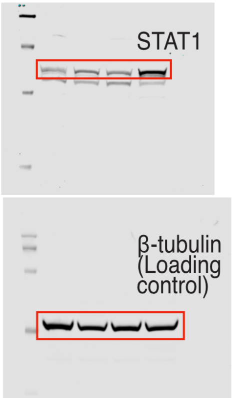

Fig 4i

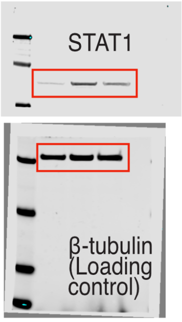

Fig 4l

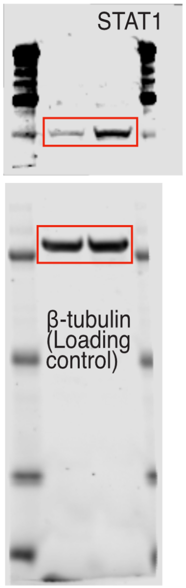

Fig 4j

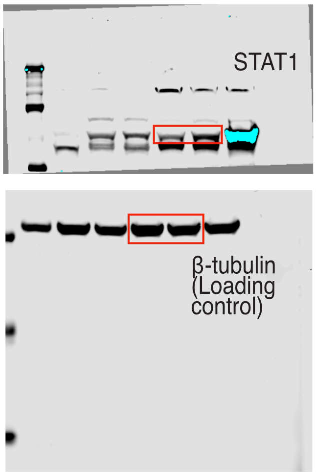

Fig 5a

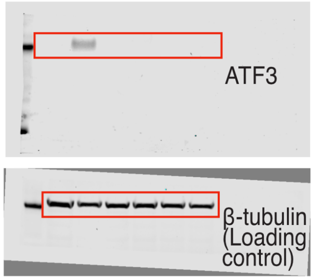

Fig 5b

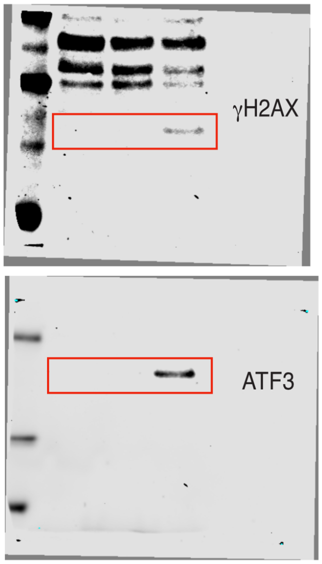

Fig 5b (continued)

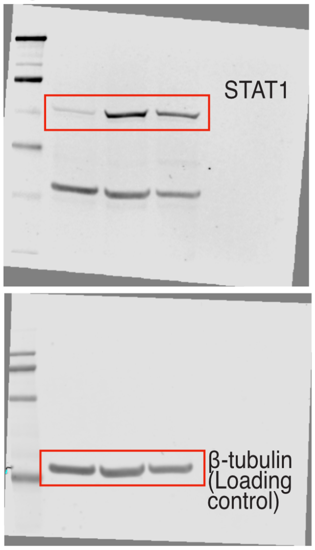

Fig 5c

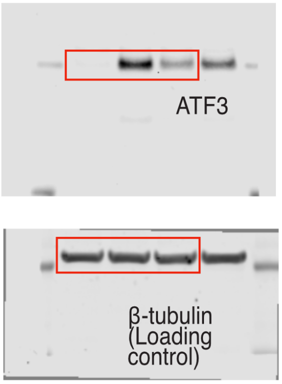

Fig 5d

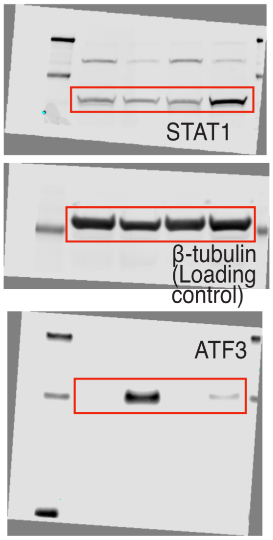

Fig 5f

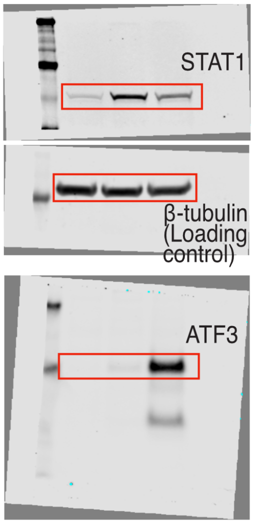

Extended Data Fig 2c

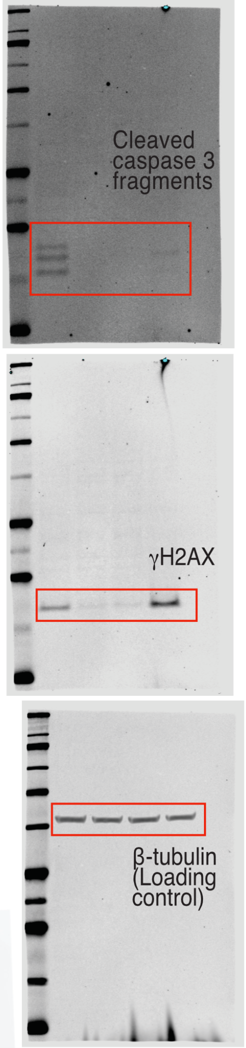

Extended Data Fig 2d

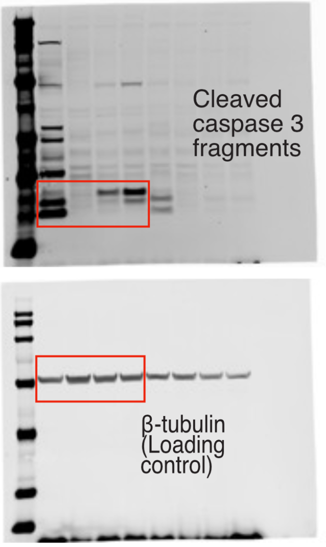

Extended Data Fig 2e

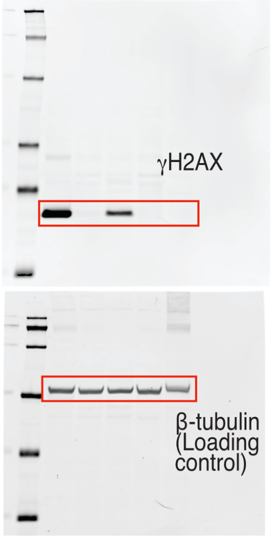

Extended Data Fig 2f

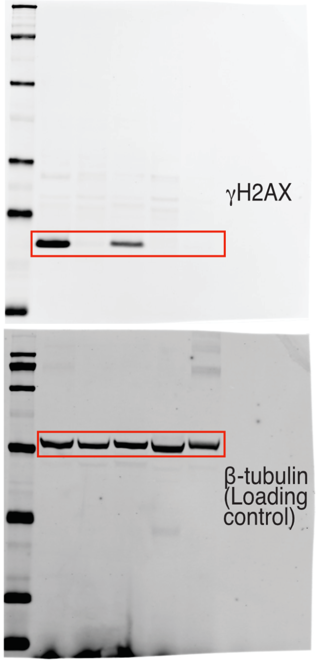

Extended Data Fig 2g

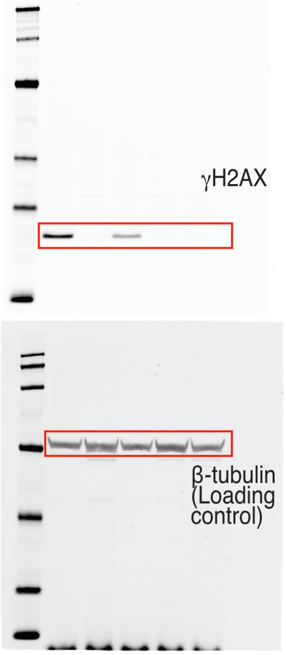

Extended Data Fig 2i

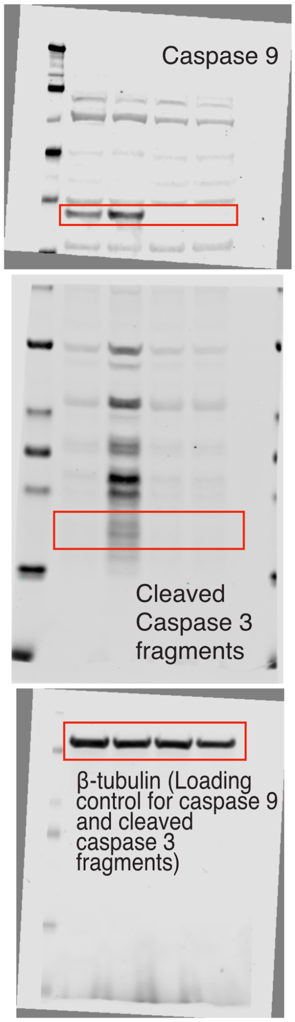

Extended Data Fig 2i (continued)

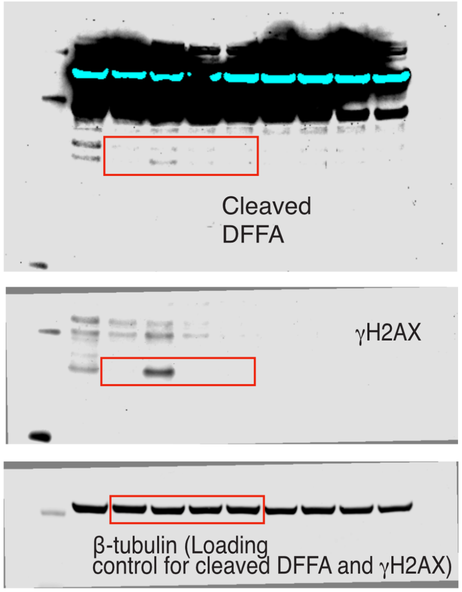

Extended Data Fig 2j

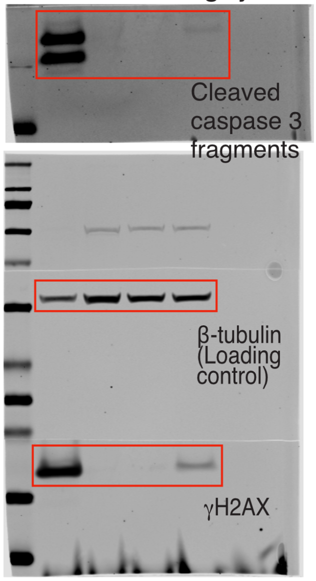

Extended Data Fig 2k

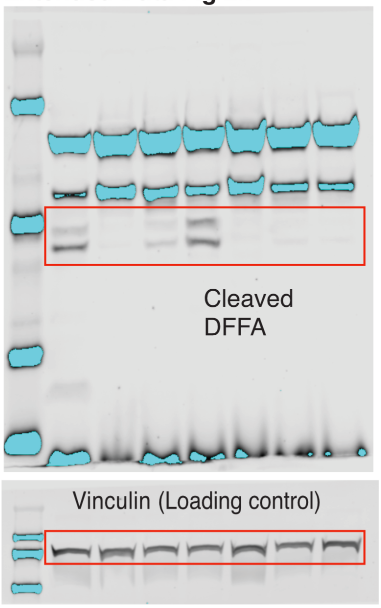

Extended Data Fig 3a

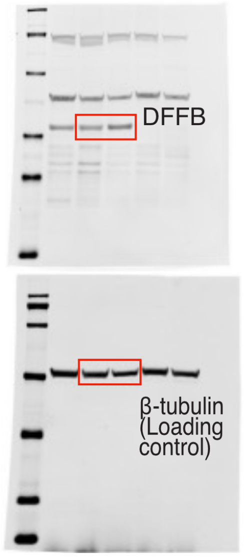

Extended Data Fig 3b

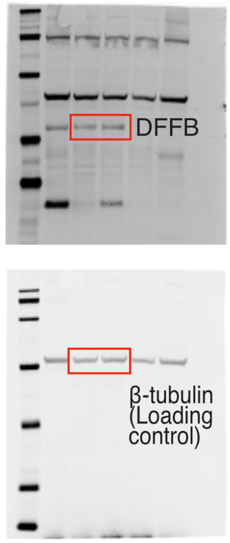

Extended Data Fig 3c

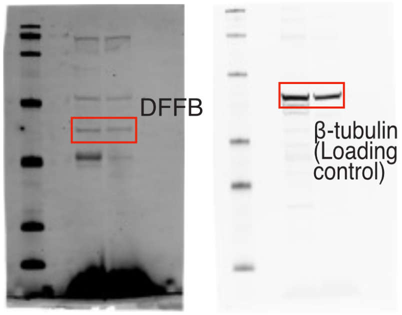

Extended Data Fig 3d

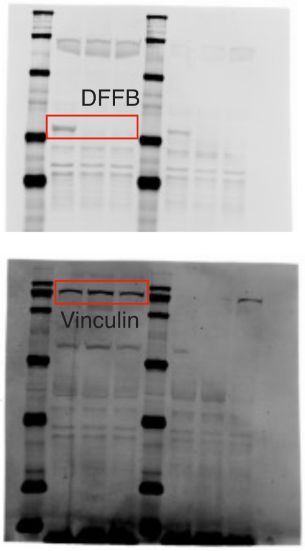

Extended Data Fig 3e

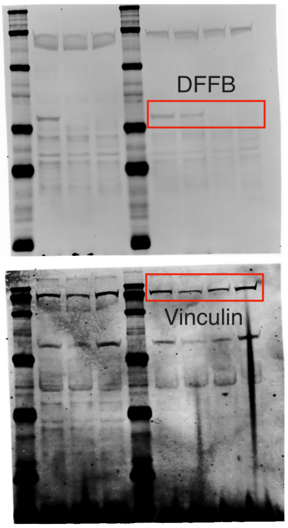

Extended Data Fig 3f

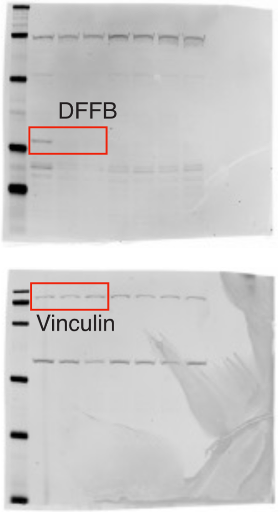

Extended Data Fig 3g

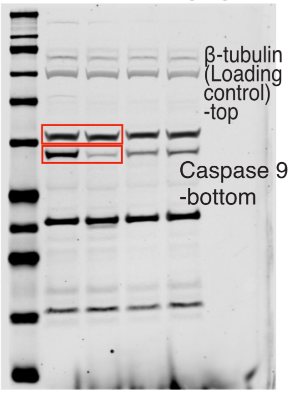

Extended Data Fig 3h

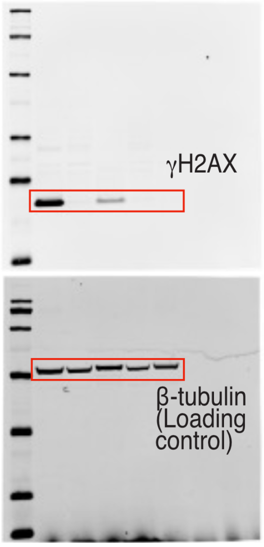

Extended Data Fig 4q

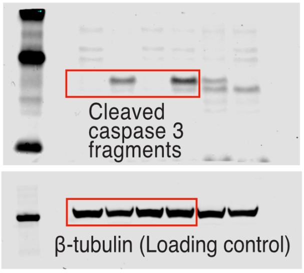

Extended Data Fig 6p

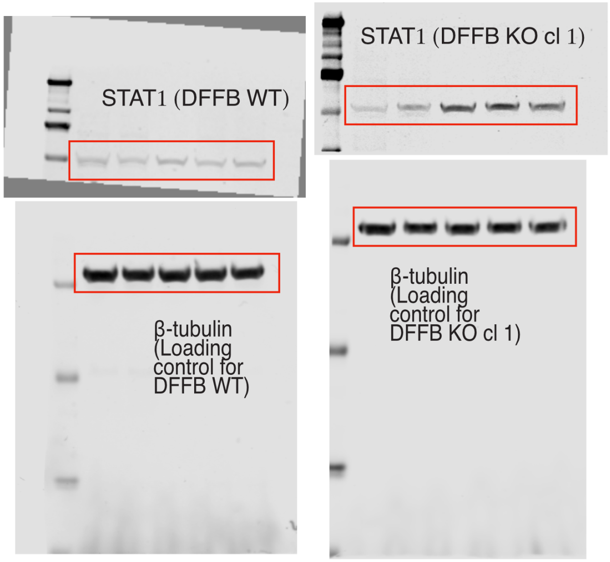

Extended Data Fig 6r

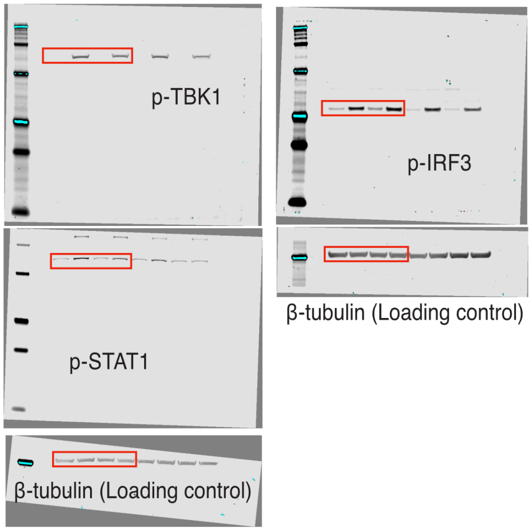

Extended Data Fig 7b

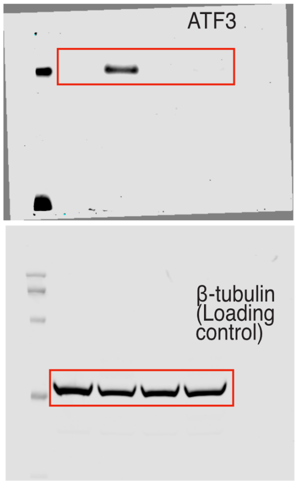

Extended Data Fig 7c

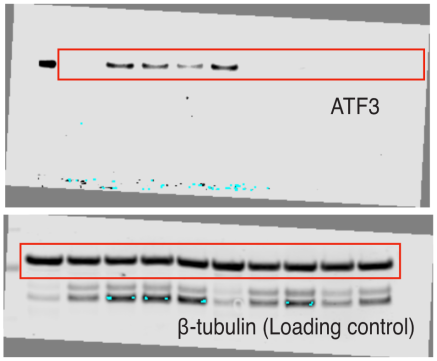

Extended Data Fig 7d

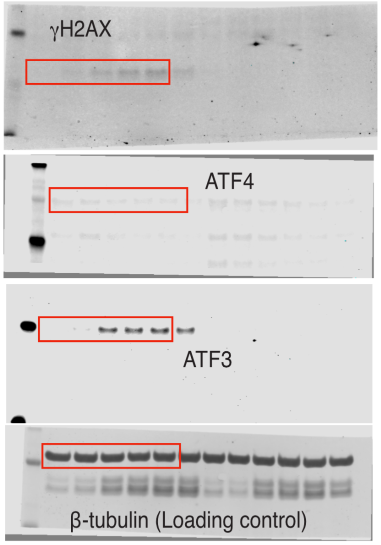

Extended Data Fig 7d (continued)

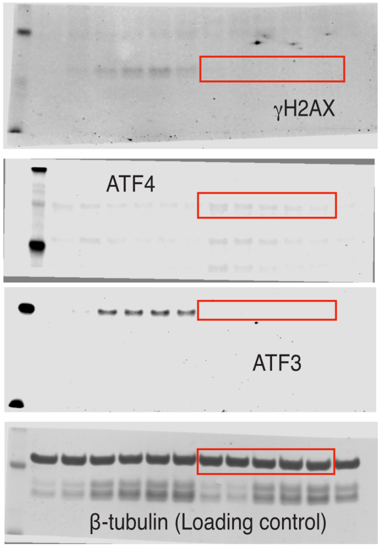

Extended Data Fig 7e

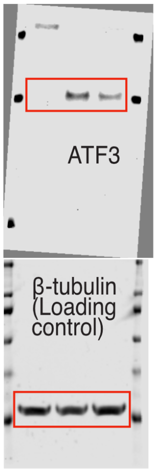

Extended Data Fig 8a

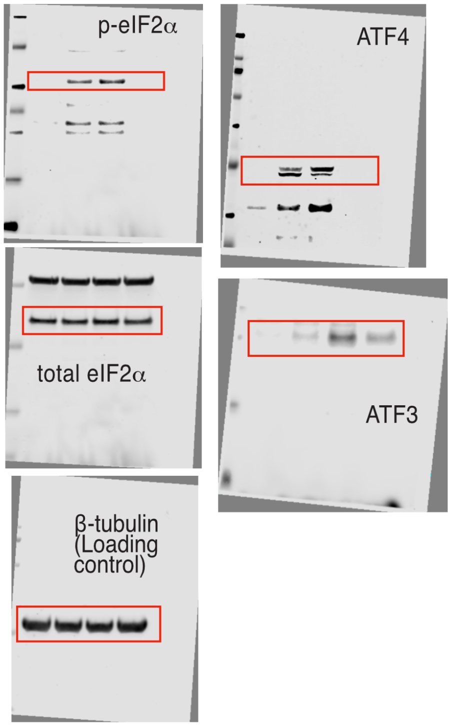

Extended Data Fig 8b

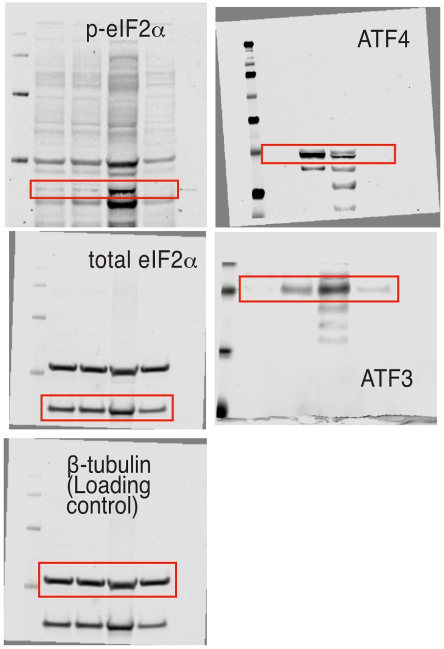

Extended Data Fig 10k

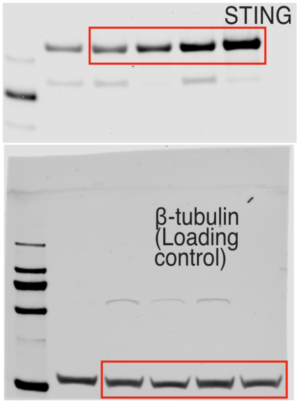

Extended Data Fig 8g

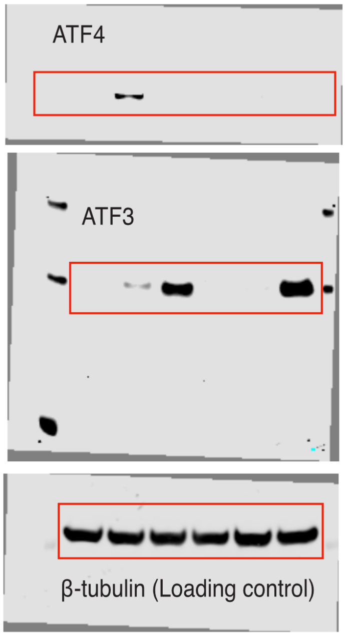

Extended Data Fig 8h

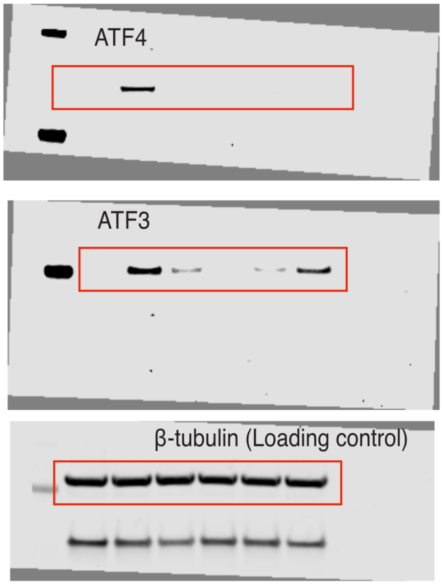

Extended Data Fig 10l

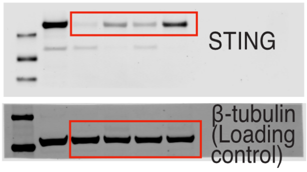

Supplement: Supplementary file 26 — Unprocessed western blots. [file 41556_2025_1810_MOESM26_ESM.pdf]
